# A role for class I p21-activated kinases in the regulation of the excitability of the actin cytoskeleton

**DOI:** 10.1242/jcs.263763

**Published:** 2025-06-23

**Authors:** Joe J. Tyler, Anthony Davidson, Megan E. Poxon, Montserrat Llanses Martinez, Pete Hume, Jason S. King, Vassilis Koronakis

**Affiliations:** ^1^ Department of Pathology, University of Cambridge, Cambridge CB2 1QP, UK; ^2^ School of Biosciences, University of Sheffield, Western Bank, Sheffield S10 2TN, UK

**Keywords:** PAK, Actin, Excitability, PI3K, Actin waves

## Abstract

The p21-activated kinases (PAKs) are involved in a range of functions, including the regulation of the actin cytoskeleton. However, although many PAK substrates identified have been implicated in the regulation of the actin cytoskeleton, a coherent picture of the total effect of PAK activation on the state of the actin cytoskeleton is unclear. Here, we show that, in mouse embryonic fibroblasts, inhibition of class I PAK kinase activity by small-molecule inhibitors leads to the constitutive production of the phosphoinositide phosphatidylinositol (3,4,5)-trisphosphate [PI(3,4,5)P_3_] on the ventral surface of the cell. The formation of patches of PI(3,4,5)P_3_ remodels the actin cytoskeleton and polarises the cell. From the overexpression of truncated and mutated PAK1 and PAK2 constructs, as well as an *in vitro* model of PAK activation, we propose that this is driven by a hyper recruitment of class I PAK and PAK-binding partners. This aberrant production of PI(3,4,5)P_3_ suggests that, by limiting its own recruitment, the kinase activity of class I PAKs acts to downregulate phosphoinositide 3-kinase (PI3K) activity, further highlighting class I PAKs as regulators of PI3K activity and therefore the excitability of the actin cytoskeleton.

## Teaser

**Highlighted Article:** Inhibition of class I p21-activated kinase activity leads to the constitutive production of PI(3,4,5)P_3_ at the plasma membrane and a dysregulation of the actin cytoskeleton.

## INTRODUCTION

The p21-activated kinases (PAKs) are a family of six serine/threonine kinases identified as acting downstream of the small GTPases Rac and Cdc42 (Manser et al., 1994, 1995). The six family members are divided by sequence homology into two subclasses, class I (PAK1, PAK2 and PAK3) and class II (PAK4, PAK5 and PAK6) (Arias-romero and Chernoff, 2008; Kumar et al., 2017). Although they share many binding partners and substrates, the PAK isoforms have different expression profiles and subcellular localisation patterns suggesting a separation of function. PAK substrates include proteins vital to the regulation of proliferation, such as Raf1 (Chaudhary et al., 1999; Zang et al., 2002), MEK1 (Parka et al., 2008) and β-catenin (Zhu et al., 2012), and as a consequence PAKs are often dysregulated during cancer progression and are considered important targets in the search for new therapeutics (King et al., 2014; Radu et al., 2014; Ong et al., 2011; Ye and Field, 2012).

All PAK isoforms contain an N-terminal p21-binding domain (PBD) and a C-terminal kinase domain (Manser et al., 1994) and adopt an autoinhibited state, mediated by an interaction between the kinase domain and the auto inhibitory domain (AID) (Zhao et al., 1998). For class I PAKs this had been thought to be due to an intermolecular interaction that results in the formation of a homodimer (Lei et al., 2000; Baskaran et al., 2012). However, recent evidence suggests that it might in fact be intramolecular, as is the case for class II PAKs (Sorrell et al., 2019). Regardless of the exact nature of the interaction, this autoinhibition is relieved by GTPase binding to the PBD domain.

Unsurprisingly for a kinase acting downstream of Rac and Cdc42, many class I PAK substrates are directly linked to the regulation of the cytoskeleton, including LIM kinase (LIMK; also known as LIMK1) (Edwards et al., 1999), filamin (Vadlamudi et al., 2003), MLC kinase (MLCK) (Sanders et al., 1999) and a component of the Arp2/3 complex, ArpC1B (Vadlamudi et al., 2004). Additionally, several kinase-independent roles for class I PAKs in the regulation of the cytoskeleton have been identified (Higuchi et al., 2008; Davidson et al., 2021) and the transient overexpression of wild-type (wt) or mutant PAK constructs, as well as the inhibition of class I PAKs by small-molecule inhibitors have all been shown to have dramatic consequences for the organisation of the actin cytoskeleton (Sells et al., 1997, 1999; Dharmawardhane et al., 2000; Kim et al., 2009; Manser et al., 1997; Itakura et al., 2013; Mierke et al., 2020). Despite the wide body of work linking PAKs to the regulation of the actin cytoskeleton, a cohesive picture of the exact role of class I PAKs in the regulation of the cytoskeleton remains elusive.

Given that the average serine threonine kinase is predicted to phosphorylate 300 unique sites, this is perhaps unsurprising (Sugiyama et al., 2019). However, the dysregulation of class I PAKs during cancer makes this an important issue, as changes to the actin cytoskeleton underpin many of the hallmarks of disease progression (Hideki and Condeelis, 2014; Olson and Sahai, 2009). Having recently identified a kinase-independent role for class I PAKs during pathogen-mediated actin remodelling (Davidson et al., 2021), we wanted to take a more general look at the consequence of PAK kinase activity on the actin cytoskeleton. We find that, in mouse embryonic fibroblasts (MEFs), the inhibition of class I PAK kinase activity drives a phosphoinositide 3-kinase (PI3K)-dependent rearrangement of the actin cytoskeleton. We show that this rearrangement depends upon the presence of PAK that lacks kinase activity but can bind to small GTPases and contains an intact AID domain. We hypothesise that this results from the maintenance of a kinase-independent scaffolding activity that is usually opposed by PAK autophosphorylation. This provides further evidence of complicated feedback mechanisms between class I PAK activation and PI3K activity, and complicates the interpretation of experiments performed with either PAK kinase inhibitors or overexpressed kinase-dead PAK constructs.

## RESULTS

### Class I PAK kinase inhibition polarises MEFs

**Fig. 1.**
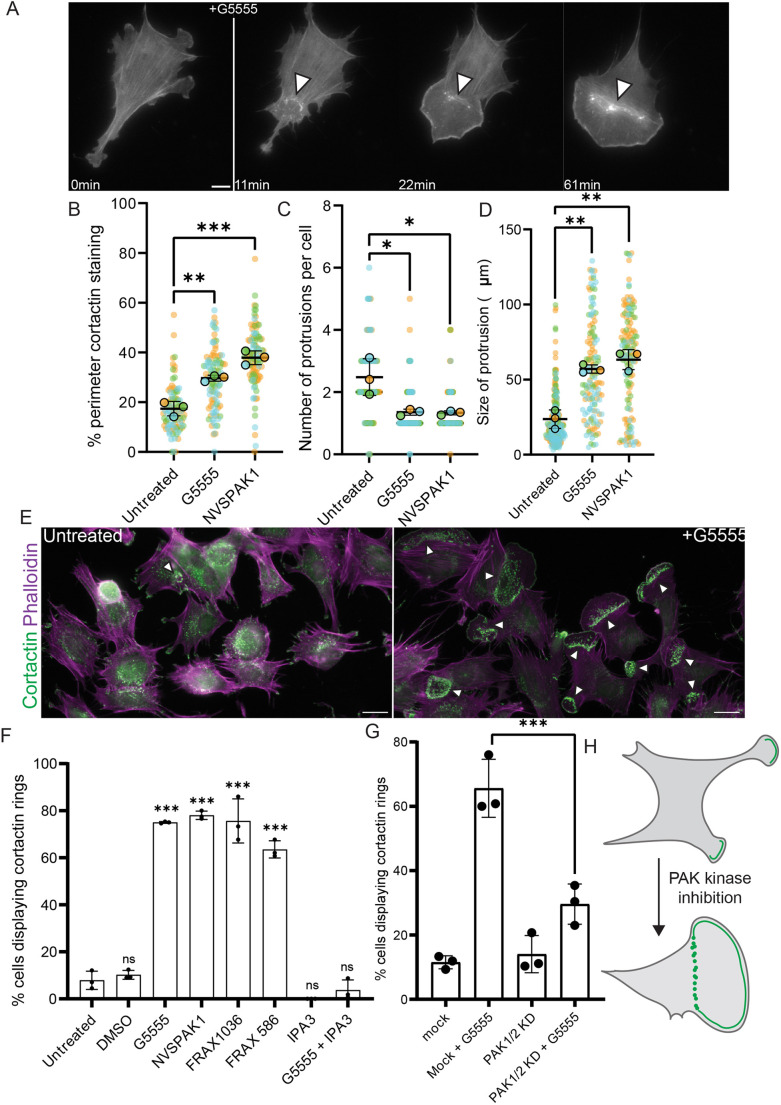
**Class I PAK kinase inhibition induces the formation of broad lamellipodia, polarising cells.** (A) Montage taken from Movie 2 of a MEF transiently overexpressing mApple–LifeAct, to mark the actin cytoskeleton, responding to the addition of 10 μM G5555. White line indicates addition of G5555; arrowheads mark actin puncta at rear of newly formed protrusion. Scale bar: 10 μm. (B–D) Quantification of organisation of the cytoskeleton and cell morphology in untreated cells (*n*=97) or in cells treated for 1 h with 10 μM of G5555 (*n*=101) or NVSPAK1 (*n*=110) from *N*=3 experiments. (B) Percentage of perimeter of cell marked by cortactin (C) Number of protrusions per cell as defined by patches of cortactin staining at the periphery of the cell. (D) Size of all individual protrusions analysed. (E) Representative images highlighting upregulation of cortactin rings following treatment for 1 h with 10 μM G5555. Scale bar: 20 μm. Arrowheads indicate cortactin/actin ring (F) Percentage of cell producing cortactin rings following treatment with various PAK inhibitors for 1 h. At least 100 cells analysed per condition for each of at least three biological repeats. All inhibitors were used at 10 μM except IPA3 which was used at 50 μM. DMSO control indicates the addition of 1 equivalent volume of DMSO. (G) Effect of knockdown of PAK1 and PAK2 expression on cortactin ring formation following treatment with 10 μM G5555 for 1 h. (H) Schematic representation of the consequence of PAK kinase inhibition in MEFs. All error bars indicate s.d. ns, no significant difference; ***P≤0.001; **P≤0.01; *P≤0.05 (ordinary one-way ANOVA followed by a post hoc Tukey's multiple comparison test).

To investigate the consequence of class I PAK kinase inhibition, we first observed MEFs before and after the addition of the ATP-competitive class I PAK kinase inhibitor G5555 by low-magnification transmitted light microscopy (Movie 1). Before the addition of the drug, cells appeared to be relatively elongated and produced multiple small, short-lived protrusions. Upon the addition of G5555, MEFs rapidly polarised, producing broad, fan-like lamellipodia which completely altered the shape of the cell. To better characterise this transition, MEFs were transiently transfected to express mApple–LifeAct to mark the actin cytoskeleton and imaged by widefield fluorescence microscopy before and following the addition of G5555. Fig. 1A is a montage taken from Movie 2, which captures the transition. Before the addition of G5555, multiple small, short-lived, protrusions could be observed. Upon addition of the drug, a new protrusion formed and steadily expanded. This protrusion was relatively stable and dramatically altered the shape of the cell as it grew.

To quantify these observations, MEFs were fixed and stained for cortactin, a marker of branched actin networks (Weed et al., 1999). Cortactin localisation to the periphery of the cell indicates an Arp2/3-dependent protrusion (Kage et al., 2022). Cells treated with G5555 showed an increase in the percentage of the perimeter that was marked by cortactin, demonstrating an increase in lamellipodia formation (Fig. 1B). Whereas untreated cells produce multiple, small protrusions, cells treated with G5555 produced on average, a single, large protrusion, highlighting a polarisation in the presence of the drug (Fig. 1C,D). A similar increase in lamellipodia production was observed in MDA-MB-231 and HAP1 cells, showing that this effect was not specific to MEFs (Fig. S1A–D). Furthermore, treatment of MEFs with the allosteric PAK1 kinase inhibitor NVSPAK1 (Karpov et al., 2015) resulted in a similar phenotype (Fig. 1B–D). DMSO alone did not lead to an increase in lamellipodia formation (Fig. S1E). Taken together, this suggests that the loss of class I PAK kinase activity promotes lamellipodia formation.

### Class I PAK kinase inhibition drives formation of cortactin rings

Upon further analysis of Movie 2, we noticed that the large protrusion that forms after addition of G5555 was marked by dynamic actin puncta, which formed a ring behind the leading edge (Fig. 1A, arrowheads). This further differentiates this structure from the smaller protrusions observed before the addition of G5555. To quantify the presence of these actin rings, we re-examined images of cells fixed and stained for cortactin and found rings of cortactin positive actin puncta were very prominent in cells treated with the class I PAK kinase inhibitors (Fig. 1E). Rings were clearly marked by cortactin, providing a convenient read out for the effect of the drug. Whereas they were only observed in 10% of untreated cells, 70–80% of cells treated with G5555 or NVSPAK1 display at least one ring of puncta containing cortactin and actin (hereafter denoted cortactin/actin rings) (Fig. 1F). A similar response was observed upon treatment with two additional ATP competitive class I PAK kinase inhibitors, FRAX 1036 and FRAX 597 (Semenova and Chernoff, 2017). To remain consistent with our previous work (Davidson et al., 2021), we used a relatively high concentration of G5555 throughout (10 μM). However, a similar response was observed upon treatment of MEFs with 1 μM G5555 (Fig. S1F). Inhibition of class I PAK kinase activity led to the upregulation of a subset of cytoskeletal structures, typified by the presence of a ring of cortactin/actin puncta.

We took advantage of these cortactin/actin puncta as a useful readout of the effect of PAK kinase inhibition to further characterise the effect of the loss of class I PAK kinase activity on the actin cytoskeleton. Like many kinases, several kinase-independent functions of class I PAKs have been previously identified (Davidson et al., 2021). Therefore, the kinase inhibitors tested so far might not block all functions of the molecule. IPA3 is a PAK inhibitor thought to block the GTPase-binding activity of class I PAKs and therefore block recruitment (Deacon et al., 2008). To determine whether a kinase-independent function of class I PAKs might contribute to the phenotype we have described, cells were treated with IPA3 and examined for the formation of cortactin/actin rings. Interestingly, IPA3 treatment alone did not drive the formation of cortactin/actin rings, whereas co-treatment of cells with G5555 and IPA3 blocked the effect of the kinase inhibitor (Fig. 1F). This suggests that the recruitment of kinase-dead class I PAK is required for the observed changes to the cytoskeleton.

To examine this further, we partially depleted PAK1 and PAK2 by siRNA treatment and checked for the formation of cortactin/actin rings (Fig. 1G). Simultaneous knockdown of PAK1 and PAK2 had no effect on ring formation in untreated cells but did impair the ability of G5555 to induce cortactin/actin rings. Quantification of knockdown efficiency is provided in Fig. S1G. This further suggests that the formation of cortactin/actin rings in the presence of kinase inhibitors is due to the local recruitment of kinase-dead PAK rather than a global reduction in total class 1 PAK activity. A schematic detailing the effect of class I PAK kinase inhibition is provided in Fig 1H.

### Effect of kinase-dead PAK1 requires GTPase binding and the AID domain

**Fig. 2.**
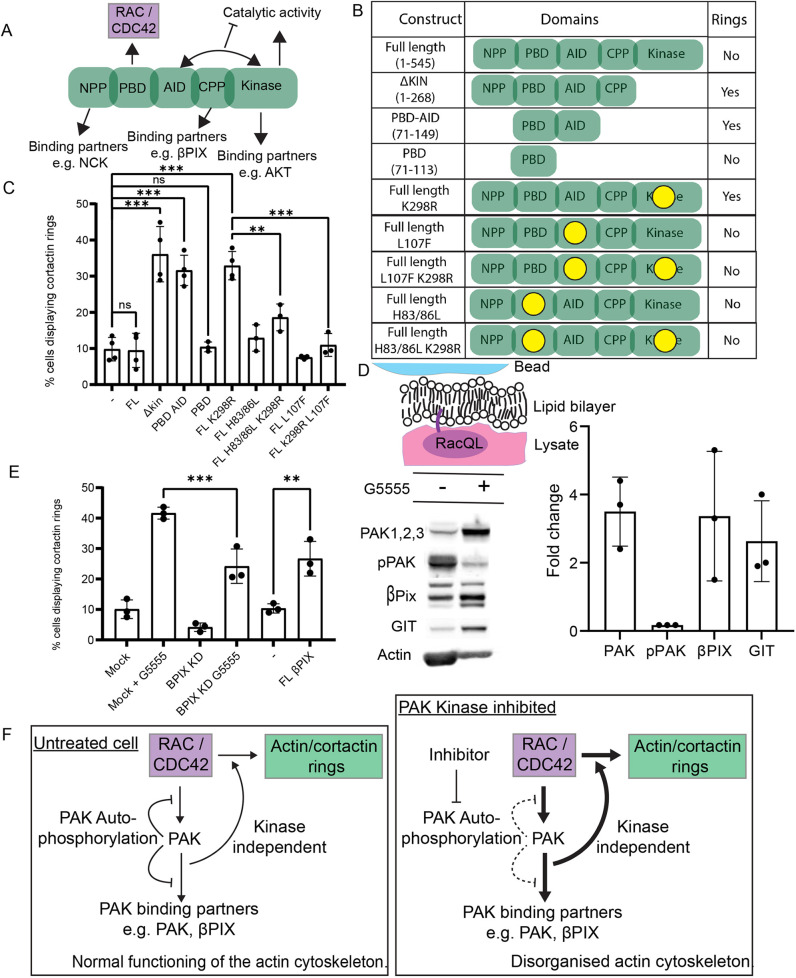
**Overexpression of dominant-negative PAK1 construct resembles treatment with PAK kinase inhibitors.** (A) Diagram schematic indicating the domain organisation of PAK1 and highlighting the localisation of a few key interactions. (B) Table indicating identity of construct expressed and summarising effect on cortactin ring formation. (C) Percentage of cells transiently expressing indicated PAK1 constructs that produce cortactin rings. At least 100 cells analysed per condition for each of at least three biological repeats. (D) Schematic describing lipid-coated bead experiment and extracts from western blots showing levels of indicated protein associated to Rac QL-coated beads following addition of 10 μM G5555 or equivalent volume of DMSO. Graph shows quantification of fold change of indicated protein or phosphorylation event as detected by western blot. (E) Percentage of cells producing cortactin rings in indicated conditions. At least 100 cells analysed per condition for each of at least three biological repeats. Where indicated, 10 μM of G5555 was added 1 h prior to fixation. (F) Schematic summary of proposed kinase dependent and independent effects on the formation of cortactin rings. All error bars indicate s.d. ns, no significant difference, ***P≤0.001; **P≤0.01 (ordinary one-way ANOVA followed by a post hoc Tukey's multiple comparison test).

To determine how class I PAK kinase inhibition influences the cytoskeleton a series of truncated PAK1 constructs were expressed in MEFs, and these cells were assessed for the presence of cortactin rings. A schematic of PAK1 highlighting key interactions is shown in Fig. 2A and results of overexpression experiments are summarised in the table (Fig. 2B).

In line with our siRNA results, simply increasing the global levels of full-length PAK1 in the cell, via the overexpression of full-length GFP–PAK1, had no effect on the formation of cortactin rings (Fig. 2C). Overexpression of a truncated construct lacking the kinase domain (GFP–PAK1 Δkin), however, led to a fourfold increase in ring formation. An example total internal reflection fluorescence microscopy (TIRFm) image of a cell coexpressing GFP–PAK1 Δkin and mCherry–LifeAct is shown in Fig. S2A, demonstrating the formation of a broad, fan-like, lamellipodia in these cells, as is seen in cells treated with class I PAK kinase inhibitors. Overexpression of a truncated PAK2 construct lacking the kinase domain (GFP-PAK2 Δkin) also led to a similar increase in ring formation (Fig. S2B,C), demonstrating that this effect is not specific to PAK1. Furthermore, overexpression of a full-length kinase-dead construct (GFP–PAK1 K298R; Brown et al., 1996) also promoted ring formation. Together, this provides further evidence that ring formation is dependent on the recruitment of class I PAK that lacks kinase activity.

### GTPase-binding is required for cortactin/actin ring formation

A further truncation consisting of the PBD and AID domain of PAK1 (GFP–PAK1 PBD-AID) promoted ring formation whereas a shorter construct consisting of just the PBD, regularly employed as a marker of active Rac (Hoppe and Swanson, 2004), did not (Fig. 2C). The PBD domain did, however, localise to the centre of actin rings in cells treated with G5555 (Fig. S2D), suggesting that it might be sufficient to recruit PAK to these structures (and that Rac and/or Cdc42 are active within the rings). To determine whether the GTPase-binding activity of this domain contributes to the activity of kinase-dead class I PAK1, we overexpressed both a GTPase-binding null full-length PAK1 (GFP–FL PAK1 H83/86L) and a GTPase-binding null kinase-dead FL PAK1 construct (GFP–FL PAK1 H83/86L K298R) and found that neither promoted ring formation (Fig. 2C). The PBD is therefore sufficient to localise PAK1 to these structures, and both the GTPase-binding activity of the PBD and the presence of the AID domain is required for the effect of kinase-dead PAK on the cytoskeleton.

### Role of the AID domain

How the AID domain might contribute to the dysregulation of the actin cytoskeleton is unclear. The AID domain of PAK binds to the kinase domain, blocking the substrate-binding pocket and holding the molecule in an autoinhibited state. To determine whether the interaction between the AID and kinase domain of PAK is required for the effect of kinase dead class I PAK, we took advantage of the L107F mutation, which has previously been shown to disrupt this interaction (Brown et al., 1996; Frost et al., 1998). Overexpression of either GFP-tagged full-length PAK1 L107F or full-length PAK1 L107F K298R did not promote the formation of cortactin/actin rings suggesting that the interaction between the AID and the kinase domain is required for the formation of these structures (Fig. 2C). Given that both GFP–PAK1 Δkin and GFP–PAK1 PBD-AID induced ring formation but lack kinase domains, we suggest that the AID domain of these constructs might be binding the kinase domain of endogenous PAK in an intermolecular interaction.

### Kinase-dead class I PAK hyperaccumulates at active Rac

As both the interaction with small GTPases and the ability to bind to more PAK is required for cortactin/actin ring formation, we hypothesised that class I PAK kinase inhibition might lead to a change in the recruitment of PAK to active GTPases. Upon activation, class I PAKs undergo a series of autophosphorylations, which weaken both the interaction between the AID and the kinase domain (Chong et al., 2001), as well as the interaction between PAK and Rac (Manser et al., 1994). In the absence of kinase activity, it is possible that the maintenance of tight GTPase binding, as well as the stabilisation of intermolecular PAK–PAK interactions, disrupts class I PAK localisation.

To test this, we used an *in vitro* model of PAK activation. Lipid-coated beads coated with prenylated constitutively active Rac1 (Rac1 Q61L; hereafter Rac QL) were incubated with pig brain lysate, in the presence or absence of G5555 and protein recruitment to the beads was assessed by western blotting (Fig. 2D). In untreated lysates, class I PAKs were pulled down by Rac1 QL and PAK phosphorylation at S144 was detected (pPAK), indicating PAK activation. Importantly, this phosphorylation was lost in the presence of G5555 showing both that it is an active process occurring in the lysate and that the drug is working in this system. Upon inhibition of class I PAK kinase activity, three times more PAK was recruited to Rac1 QL beads. Loss of class I PAK kinase activity therefore leads to the aberrant accumulation of PAK, suggesting that one consequence of class I PAK kinase activity is to limit recruitment to sites of small GTPase activation (Fig. 2D).

### Role of βPix during PAK inhibition

We next asked how accumulation of kinase dead class I PAK at small GTPases influences the organisation of the cytoskeleton. We hypothesised that enrichment of PAK might lead to the accumulation of PAK-binding partners. βPix (also known as ARHGEF7) is a Rac and Cdc42 GEF that binds to class I PAKs and is therefore a good candidate to mediate changes to the actin cytoskeleton (Manser et al., 1998). Western blot analysis showed that both βPix and its constitutive binding partner GIT1 were enriched on Rac QL beads in the presence of the G5555 (Fig. 2D). In agreement with a potential role for βPix during class I PAK kinase inhibition, treatment of cells with siRNA targeting βPix led to a 50% reduction in the number of cells that generated cortactin/actin positive rings upon treatment with G5555 (Fig. 2E). We were, however, unable to detect βPix in MEF extract and so were unable to validate the efficiency of any reduction in protein level. To further confirm a role for βPix in this process we transiently overexpressed βPIX–GFP and checked for cortactin/actin ring formation. Upon overexpression of this construct there was a 2.5-fold increase in the number of cells making rings (Fig. 2E).

Overall, βPix is required for the full response of the cell to class I PAK kinase inhibition and overexpression of full-length βPix alone is sufficient to mimic the phenotype of PAK inhibition. We therefore conclude that the dramatic rearrangements of the actin cytoskeleton upon class I PAK kinase inhibition depends upon the presence of a molecule of PAK that lacks catalytic activity but maintains the GTPase-binding activity of the PBD domain and the kinase-binding activity of the AID domain. This results in the hyperaccumulation of class I PAK and PAK-binding partners and the dysregulation of the actin cytoskeleton (Fig. 2F).

### Cortactin puncta resemble podosomes

**Fig. 3.**
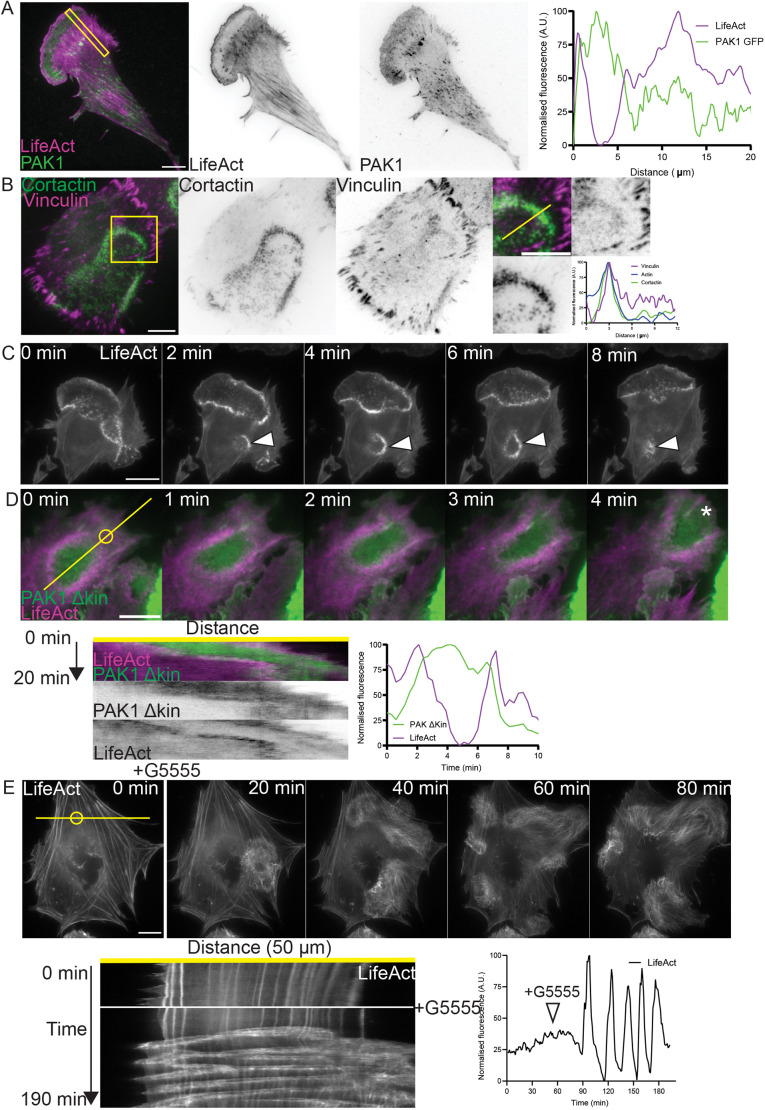
**Rings of cortactin/actin puncta resemble actin waves.** (A) TIRFm image of a cell coexpressing GFP–PAK1 FL and mApple–LifeAct and treated with 10 μM G5555 for at least an hour, demonstrating localisation of PAK1 to the centre of ring structure. (B) Representative image of cell stained for vinculin (green) and cortactin (magenta) showing localisation of vinculin to the ring of cortactin puncta. (C) Montage taken from Movie 3 of cell transiently overexpressing mApple–LifeAct, treated with 10 μM G5555 for at least an hour, highlighting dynamics of internal rings. (D) Montage taken from Movie 4 of MEF expressing GFP PAK1 Δkin and mApple LifeAct. Accompanying kymograph taken from line indicated in the first panel. Graph indicates fluorescence intensity overtime in region indicated by circle in first panel. (E) Montage taken from Movie 5 of a B16F1 cell transiently overexpressing mEGFP–LifeAct responding to the addition of 10 μM G5555. Accompanying kymograph generated from the same movie highlighting the disruption of the actin cytoskeleton upon addition of G5555 taken from line indicated in first panel. Graph indicates fluorescence intensity over time in region of the cell marked by a circle in first panel. Scale bars: 10 μm. A.U., arbitrary units.

To determine how the recruitment of a kinase-dead molecule of PAK might influence the cytoskeleton, we set out to better define the cortactin/actin rings observed in Fig. 1E. First, we asked where PAK1 localised. Live-cell TIRFm of cells expressing GFP–PAK1 and mApple–LifeAct, and treated with G5555, showed PAK1 to localise within the actin ring and at focal adhesions across the ventral surface of the cell (Fig. 3A). A similar localisation was observed for endogenous class I PAKs by widefield fluorescence microscopy following immunolabelling of class I PAKs (Fig. S3A).

Next, we further probed the molecular composition of the cortactin/actin rings. A combination of live-cell imaging of overexpressed fluorescent markers and immunostaining of endogenous proteins was used to dissect the molecular composition of the cortactin-positive actin puncta. Unsurprisingly, these were shown to contain a component of the Arp2/3 complex (Fig. S3B). They were also marked by the nucleation-promoting factor N-WASp (also known as WASL; Fig. S3C) and Cyfip, a component of the wave regulatory complex (Fig. S3D), as well as Myo1e (Fig. S3E).

The size and molecular composition of these puncta are reminiscent of podosomes. These are actin-dependent adhesive structures characterised by a dense actin core surrounded by a ring of adaptor proteins, such as vinculin (van den Dries et al., 2019). Immunostaining of endogenous vinculin revealed a diffuse localisation around the actin puncta, suggesting that they might indeed be adhesive (Fig. 3B). These puncta therefore appear similar in both superficial appearance and molecular composition to podosomes, but they lack the clear organisation of the actin core and adhesive ring. We therefore tentatively term these structures ‘podosome like’.

### Cortactin rings resemble actin waves, suggesting an increase in excitability of the actin cytoskeleton

These ‘podosome-like’ puncta are organised into higher-order structures, referred to here as cortactin/actin rings, that are themselves dynamic. To gain a better understanding of the identity of this superstructure we further examined time lapse movies of cells expressing mApple–LifeAct following treatment with G5555 (Fig. 3B; Movie 3). Fig. 3C highlights the dynamic nature of these structures. The ring of actin puncta can be seen to fluctuate in place, dramatically growing and shrinking. The actin in the centre of this ring appears different in organisation to the actin outside and where it contacts the edge of the cell it drives the formation of a protrusion.

Such dynamics are not typical of classic lamellipodia and are more reminiscent of those described for actin teeth in macrophages performing stalled phagocytosis or the wavefronts of actin observed during circular dorsal ruffle (CDR) formation (Barger et al., 2019; Ostrowski et al., 2019; Bernitt et al., 2015). Indeed smaller rings can be observed forming within and travelling across the body of the cell (Fig. 3C, arrowheads), similar to the actin waves observed in lab strains of *Dictyostelium* (Vicker, 2002b; Bretschneider et al., 2009) as well as in certain mammalian cell types (Vicker, 2002a; Zhan et al., 2020). Changes in in the signalling networks that organise these structures have been shown to influence protrusion dynamics and cell shape (Miao et al., 2017). We therefore propose that the polarisation observed in MEFs might be driven by an increase in the excitability of the actin cytoskeleton.

These dynamics are further highlighted in Movie 4 and Fig. 3D, a montage from TIRFm imaging of a cell expressing mApple–LifeAct and GFP–PAK1 ΔKin. Here, a patch of PAK1 ΔKin is seen surrounded by a dynamic ring of actin. As the patch moves so does the ring, until it contacts the periphery of the cell and drives the formation of a protrusion (Fig. 3D, indicated by * in final panel). The correlation of the PAK1 ΔKin patch and actin ring is highlighted in the accompanying kymograph, which was generated along the line indicated in the first panel of Fig. 3D. The accompanying graph shows fluorescence intensity over time within the region marked with a circle in the first panel of Fig. 3D. This further shows how the actin cytoskeleton remodels as the patch of kinase-dead PAK travels across the ventral surface of the cell.

Finally, we wanted to determine whether similar dynamics could be observed in another cell line. B16F1 cells are routinely used in studies of the actin cytoskeleton for a number of reasons, not least because they form prominent lamellipodia (Kage et al., 2022; Buracco et al., 2024). We hypothesised that the apparent excitability of the cytoskeleton in this cell line might make for a prominent response to class I PAK kinase inhibition. B16F1 cells were therefore transiently transfected to express eGFP–LifeAct and observed via widefield fluorescence microscopy before and then during the addition of G5555 (Fig. 3E; Movie 5). Before addition of the drug the cytoskeleton of the cell appeared relatively stable with prominent stress fibres and only small, short-lived protrusions. Following the addition of G5555 an actin wave could be seen to form that begun to traverse around the cell. This wave split at multiple points until many waves could be seen travelling around the cell, driving protrusion where they contacted the cell edge and reorganising the prominent stress fibres where they passed. The dramatic change in the state of the cytoskeleton is highlighted in the accompanying kymograph, as well as a read out of actin intensity overtime at the region of the cell marked with a circle in the first panel.

### Lipid composition of rings

Actin waves and related structures are organised around regions of the plasma membrane with a specific identity, within which phosphatidylinositol (3,4,5)-trisphosphate [PI(3,4,5)P_3_] and phosphatidylinositol (3,4)-bisphosphate [PI(3,4)P_2_] are enriched and phosphatidylinositol (4,5)-bisphosphate [PI(4,5)P_2_] is depleted (Zhan et al., 2020; Masters et al, 2016; Quinn et al., 2021; Ostrowski et al., 2019; Gerhardt et al., 2014). To examine whether a similar organisation of lipid species might organise the cortactin/actin ring structures observed upon inhibition of class I PAK kinase activity, fluorescent markers of various phosphoinositides were expressed in MEFs expressing mApple–LifeAct to mark the actin cytoskeleton. These cells were then treated with G5555 and the localisation of the different markers relative to the actin cytoskeleton compared using TIRFm.

**Fig. 4.**
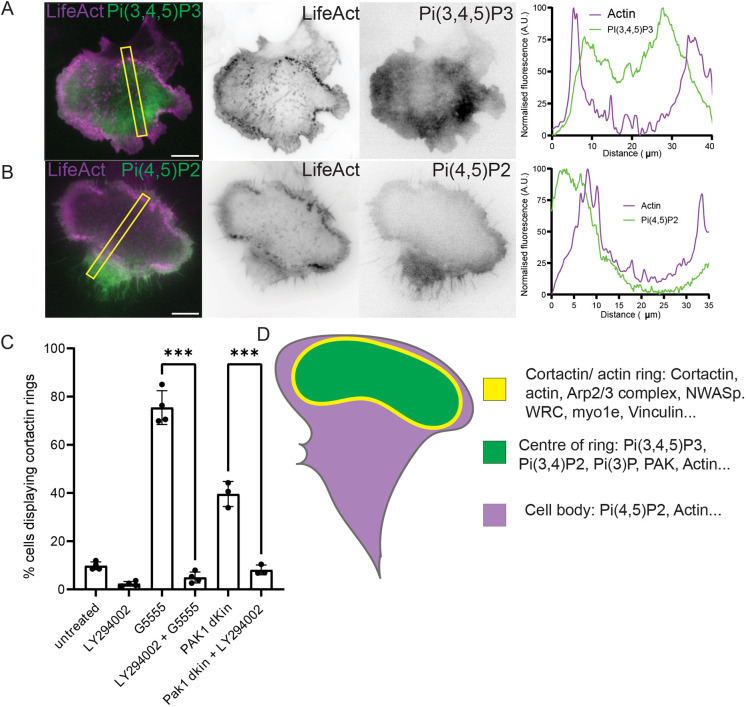
**Formation of cortactin/actin rings is PI3K dependent.** (A) Still from Movie 6 of an MEF transiently overexpressing mCherry–LifeAct and PI(3,4,5)P_3_ marker (GFP BTK PH) following treatment with 10 μM G5555 for at least an hour. Accompanying linescan demonstrates localisation PI(3,4,5)P_3_ marker relative to actin cytoskeleton. (B) Still from Movie 7 of an MEF transiently overexpressing mEGFP–LifeAct and PI(4,5)P_2_ marker (mCherry PH domain of PLCdelta1) following treatment with 10 μM G5555 for at least an hour. Accompanying linescan demonstrates localisation of PI(4,5)P_2_ marker relative to actin cytoskeleton. (C) Percentage of cells producing cortactin rings in the presence and absence of 10 μM PI3K inhibitor LY294002 alongside either cotreatment with 10 μM G5555 or overexpression of GFP–PAK1Δkin. At least 100 cells analysed per condition for each of at least three biological repeats. Inhibitors were added 1 h before fixation. (D) Schematic summarising the localisation of various proteins upon addition of G5555. Scale bars: 10 μm. All error bars indicate s.d. ***P≤0.001 (ordinary one-way ANOVA followed by a post hoc Tukey's multiple comparison test). A.U., arbitrary units.

After treatment with G5555 the PI(3,4,5)P_3_ marker (GFP BTK PH) was observed to localise within the ring of actin puncta (Fig. 4A; Movie 6). BTK fluorescence dropped off sharply outside of the ring and there was no corresponding peak of PI(3,4,5)P_3_ observed relative to the actin puncta. Interestingly the PI(3,4)P_2_ marker (GFP–TAPP1 PH) and a phosphatidylinositol 3-phosphate [PI(3)P] marker (EYFP–p40phox px domain) also localised within the actin ring (Fig. S4A,B). The PI(4,5)P_2_ marker (mCherry–PLC PH) was clearly excluded from the centre of the ring of actin puncta (Fig. 4B; Movie 7).

Cortactin/actin rings therefore form around patches of membrane enriched in PI(3,4,5)P_3_, PI(3,4)P_2_ and PI(3)P. Owing to the prevalence of 3′ phosphorylated phosphoinositides within the centre of the rings, we wanted to determine the role of PI3Ks on the formation of these structures. Co-treatment with the PI3K inhibitor LY294002 abolished cortactin ring formation in both cells treated with G5555 and cells expressing PAK1 Δkin (Fig. 4C). The formation of these structures upon class I PAK kinase inhibition is therefore PI3K dependent. In agreement with this, we detected an increase in the ratio of phosphorylated AKT family proteins to non-phosphorylated AKT family proteins (pAKT:AKT) by western blot in cells treated with G5555, consistent with a global increase in PI(3,4,5)P_3_ (Fig. S4C). We propose then that the accumulation of PAK upon PAK kinase inhibition ultimately leads to a dysregulation of PI3K signalling and the constitutive production of PI(3,4,5)P_3_ domains on the plasma membrane. These domains organise actin polymerisation resulting in the formation of rings of cortactin/actin positive puncta, which travel across the plasma membrane (Fig. 4D).

### PI3K dependence of phenotypic changes upon class I PAK kinase inhibition

**Fig. 5.**
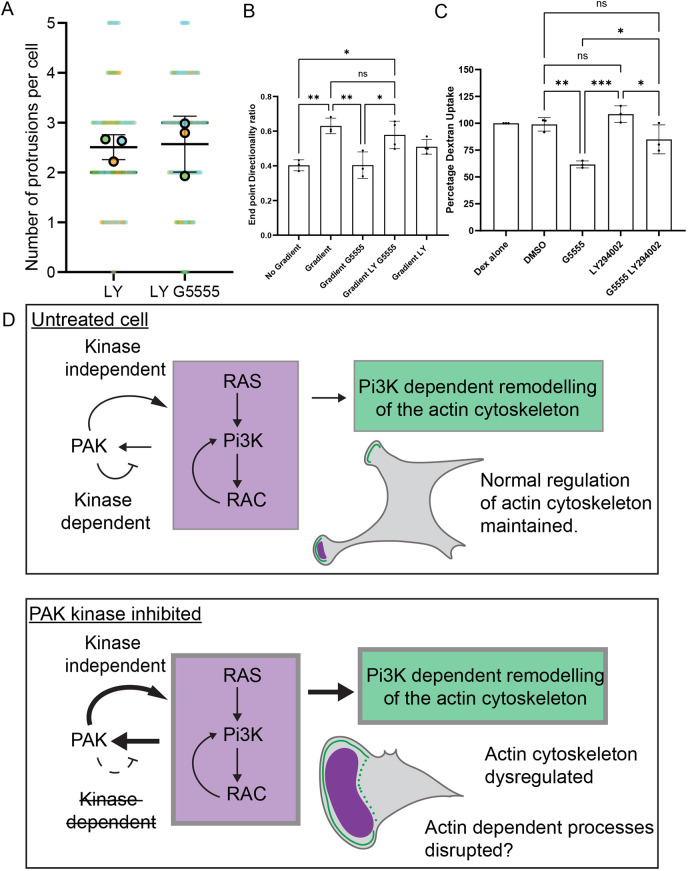
**PI3K dependence of phenotypic changes upon PAK kinase inhibition.** (A) Quantification of the number of protrusions per cell as defined by patches of cortactin staining at the periphery of the cell in cells treated with 10 μM LY294002 (*n*=97) or co-treated with 10 μM LY294002 and 10 μM G5555 (*n*=108) for an hour. From *N*=3 experiments. (B) End point directionality ratio of cells tracked over a period of 16.5 h migrating in chemotaxis chambers in indicated conditions. An hour was left between the initiation of the assay and the start of tracking to allow cells to respond to inhibitors. All inhibitors were used at 10 μM. At least 30 cells analysed per condition for each of at least three biological repeats. (C) Relative dextran uptake in MiaPacca cells in indicated conditions measured by flow cytometry. Dextran uptake normalised to untreated levels. All inhibitors were used at 10 μM. Cells were pretreated with inhibitor for 30 min, after which they were co-incubated with indicated drug and 0.25 mg/ml lysine fixable TMR conjugated 70 kDa dextran for 90 min. At least 10,000 cells were counted per condition, per repeat. (D) Schematic summarising proposed mechanism by which PAK kinase inhibition disrupts the regulation of the actin cytoskeleton. All error bars indicate s.d. ns, no significant difference, ***P≤0.001; **P≤0.01; *P≤0.05 (ordinary one-way ANOVA followed by a post hoc Tukey's multiple comparison test).

PI(3,4,5)P_3_ is produced at the plasma membrane in response to acute external stimulation (Karunarathne et al., 2013). This is one mechanism by which the actin cytoskeleton can be organised in response to a change in the external environment of the cell. The aberrant production of PI(3,4,5)P_3_ in the presence of class I PAK kinase inhibitors might therefore mimic the application of an external signal. This might explain the polarisation of the cytoskeleton in the absence of an external directional cue, described in Fig. 1. We therefore further analysed the localisation of cortactin in cells treated with LY294002 or co-treated with LY294002 and G5555. Although cells treated with LY294002 still produced lamellipodia, no increase in lamelipodia formation was observed upon co-treatment with G5555 (Fig. S5A,B). Furthermore, there was no change in the number of protrusions formed per cell demonstrating that MEFs treated with G5555 do not polarise in the absence of PI3K activity (Fig. 5A).

### Migration

We hypothesised that this PI3K-dependent polarisation of the cytoskeleton might disrupt the ability of the cell to respond to external signals as the pathway could already be saturated. To test this, we analysed the effect of PAK kinase inhibition on cells migrating randomly and in the presence of a chemotactic gradient. Class I PAKs have previously been implicated in the regulation of cell migration, with both overexpression of mutant constructs and inhibition with small-molecule inhibitors having been shown to influence both the speed and directionality of migrating cells (Itakura et al., 2013; Sells et al., 1999). The aberrant over-production of PI(3,4,5)P_3_ has also been shown to disrupt efficient cell migration (Veltman et al., 2014).

Upon the addition of a chemotactic gradient, untreated cells responded by orientating migration along the gradient. One read out of this change was an increase in the directional persistence, as cells turned less as their migration became directed (Fig. 5B). Upon the addition of G5555, however, MEFs no longer increased directional persistence in the presence of a chemotactic gradient. However, co-treatment with LY294002 restored the ability of cells treated with G5555 to migrate in a more persistent manner within a chemotactic gradient. Plots displaying a random subset of cell trajectories are displayed in Fig. S5C. This suggests that the aberrant production of PI(3,4,5)P_3_ in the absence of class I PAK kinase activity disrupts the ability of the cell to respond to external signals. This does not mean that the dysregulation of PI3K accounts for all effects of class I PAKs in cell migration but demonstrates that directional migration is possible in the absence of PAK kinase activity if PI3K activity is also inhibited.

### Macropinocytosis

We finally wanted to determine whether the dramatic rearrangement of the cytoskeleton observed upon PAK kinase inhibition could disrupt other actin-dependent processes. Macropinocytosis is the bulk uptake of extracellular fluid and requires an actin dependent reorganisation of the plasma membrane. Overexpression of various mutant PAK constructs, treatment with PAK kinase inhibitors and knockdown of PAK1 has previously been shown to influence levels of macropinocytosis (Lee et al., 2019; Dharmawardhane et al., 2000; Nazemi et al., 2024). We therefore wanted to determine whether the PI3K-dependent rearrangement of the cytoskeleton might explain some of the effect of class I PAK kinase inhibition on this process.

Given that macropinocytosis is PI3K dependent in many cell types, we used MiaPaCa cells, which constitutively perform PI3K-independent macropinocytosis (Kim et al., 2022). We first examined the response of MiaPaCa cells to treatment with G5555. Although we did not observe the formation of cortactin/actin rings, a PI3K-dependent change in cell spreading was observed upon treatment with G5555 (Fig. S5D,E) consistent with an increase in the excitability of the cytoskeleton (Miao et al., 2017). We next examined the effect of PAK inhibition on macropinocytosis, as measured by the uptake of 70 kDa dextran (Fig. 5C). As expected, untreated MiaPaCa cells performed macropinocytosis and this was unaffected by the addition of LY294002. Addition of G5555, however, led to a nearly 50% reduction in uptake. Amazingly, this effect was partially reversed by co-treatment with LY294002. This further demonstrates that the aberrant formation of PI(3,4,5)P_3_ in response to class I PAK kinase inhibition can disrupt the normal functions of the actin cytoskeleton (Fig. 5D). Again this does not mean that dysregulated PI3K signalling accounts for all effects of class I PAKs during micropinocytosis, as several studies have shown an effect on macropinocytosis following a knockdown of PAK (Nazemi et al., 2024; Martinez et al., 2024) but clearly demonstrates that caution is required when attributing phenotypes to the loss of PAK kinase activity when using PAK kinase inhibitors alone.

## DISCUSSION

Previous studies have reported marked effects on the cytoskeleton upon both the overexpression of PAK and mutant PAK constructs as well as treatment with small-molecule inhibitors. In this study, we quantify and further characterise an increase in lamelipodia formation upon inhibition of class I PAK kinase activity in MEFs, as well as in several other cell lines. We show that this correlates with an increase in the presence of a subset of PI3K-dependent actin structures. We conclude that class I PAK kinase inhibition results in a dysregulation of PI3K activity and that this explains a number of the phenotypic changes observed upon PAK kinase inhibition. We hypothesise that this is due to the disruption of a kinase-dependent negative feedback loop, which in untreated cells limits the recruitment of class I PAK and PAK-binding partners to sites of GTPase activation. Together, this provides further evidence of feedback from class I PAK kinase activation to PI3K activity and positions class I PAKs and PAK-binding partners as vital nodes in the regulation of the excitable pathways that control PI3K signalling.

Numerous studies of class I PAKs have demonstrated effects on the actin cytoskeleton upon overexpression of various mutant constructs as well as inhibition of kinase activity by small-molecule inhibitors. We find that the formation of PI3K-dependent cortactin/actin rings only occurs when PAK kinase activity is inhibited. Importantly, we show that kinase inhibition by small-molecule inhibitors recapitulates the effect of the overexpression of kinase-dead PAK constructs. This suggests that the effect of kinase-dead constructs represents the genuine inhibition of endogenous PAK kinase activity and demonstrates that similar phenotypes can be driven at endogenous levels of PAK expression.

The model proposed in Fig. 2F suggests that (1) the recruitment of class I PAKs and PAK-binding partners modulates the actin cytoskeleton in a kinase-independent manner, and (2) that PAK kinase activity limits the total accumulation of PAK to sites of small GTPase activation. In the presence of PAK kinase activity, recruitment of PAK to small GTPases is therefore self-limiting and an optimal level of PAK recruitment, and therefore PAK scaffolding activity, is achieved. It is interesting to speculate that levels of PAK recruitment might be optimally tuned to elicit an appropriate cytoskeletal response following small GTPase activation. It will be important in the future to better characterise this scaffolding activity in the presence of PAK kinase activity. For example, in the absence of PAK as a scaffold, are cells in which levels of class I PAKs have been lowered by knockdown or knockout able to organise a coherent response to an external signal?

The mechanism linking hyper recruitment of class I PAKs and PAK-binding partners to PI3K activation also requires further investigation. We show that βPix is required for the full response of the cell to PAK kinase inhibition, but how βPix mediates this response is uncertain. Furthermore, class I PAKs and the βPix–GIT complex interact with multiple components of the excitable pathway that regulates PI(3,4,5)P_3_ production including binding directly to PI3K (Papakonstanti and Stournaras, 2002), Rac (Manser et al., 1994), ERK (Yin et al., 2005) and AKT (Higuchi et al., 2008), as well as phosphorylating the Ras effector Raf1 (Chaudhary et al., 1999) and the tumour suppressor Merlin (NF2) (Xiao et al., 2002). The consequences of these varied activities and how they are integrated into a coherent response upon PAK recruitment and activation is far from clear. Owing to the complex nature of interactions accounted for by the PAK–βPix–GIT complex, the accumulation of these proteins is likely to be complicated and perhaps non-linear (Zhu et al., 2020; Premont et al., 2004).

Regardless of the exact mechanism, in MEFs the loss of class I PAK kinase activity results in the production of ventral PI(3,4,5)P_3_ domains implying a dysregulation of the excitable networks that regulate PI3K activity. Acute perturbations to this pathway have previously been shown to drive dramatic switches in cell shape in both *Dictyostelium* and mammalian cell lines in agreement with the polarisation observed in MEFs (Miao et al., 2017; 2019; Devreotes et al., 2017; Zhan et al., 2020; Pal et al., 2023; Kuhn et al., 2024 preprint). This positions class I PAKs as a potential node at which the excitable pathways controlling PI3K activity might be regulated. Again, it will be important now to test whether the opposite is true – do increased levels of PAK kinase activity negatively regulate the production of PI(3,4,5)P_3_? Partial depletion of PAK1 and PAK2 by siRNA did not in itself promote the formation of cortactin/actin rings but it would be interesting to test this further in a system with higher basal PI3K activity.

PI(3,4,5)P_3_ domains organise the cytoskeleton across large regions of both time and space and facilitate macropinocytosis and phagocytosis, as well as a subset of protrusions in a range of cell lines (Veltman et al., 2016; Lutton et al., 2023; Ostrowski et al., 2019). The presence of these structures has been correlated with oncogenic transformation (Zhan et al., 2020) and they have recently been implicated in the regulation of glycolysis (Zhan et al., 2024 preprint). They have been studied extensively in *Dictyostelium*, where lab strains constitutively produce ventral PI(3,4,5)P_3_ domains that are amenable to imaging via TIRFm (Gerhardt et al., 2014). Analogous ventral structures can be induced in a range of mammalian cell types; however, comparison between mammalian and *Dictyostelium* models has been somewhat limited by the relative difficulty of culturing and transfecting the specialised cell lines required as well as due to the transient nature of the response of the mammalian system to stimulation. The dramatic and long-lasting induction of ventral PI(3,4,5)P_3_ domains in MEFs and B16F1 cells therefore provides a convenient model to better characterise the organisation and dynamics of these structures in mammalian cell lines.

Finally, the rapid formation of large PI(3,4,5)P_3_ domains following the inhibition of class I PAK kinase activity complicates the interpretation of results obtained in the presence of class I PAK kinase inhibitors. The formation of PI(3,4,5)P_3_ at the plasma membrane necessarily influences many signalling pathways (Swanson et al., 2019), and PI(3,4,5)P_3_ domains organise many components of the cytoskeleton, potentially limiting the availability of key factors. Furthermore, they dramatically remodel the cytoskeleton, specifically the ventral cortex. The formation of these structures, which at times encompass much of the ventral surface of the cell, necessarily influences the mode of adhesion as well as the contractility of the cell. This in turn might further influence the state of key signalling pathways as well as the physical properties of the cell itself. Together, this makes it hard to uncouple direct effects of the loss of class I PAK kinase activity from indirect effects stemming from the modulation of PI3K signalling and provides further justification for the recent development of a PAK1-selective degrader (Chow et al., 2023).

## MATERIALS AND METHODS

### Cell culture

MEFs, B16F1s (ATCC), MiaPaCas and MDA-MB-231 were maintained in Dulbecco's Modified Eagle Medium (DMEM) supplemented with 10% FBS and 100 U/ml penicillin-streptomycin (complete medium; Thermo Fisher Scientific). MEFs were a kind gift from Dr Leszek Kotula (Department of Urology, Biochemistry & Molecular Biology, SUNY Upstate Medical University, USA); MiaPaCa cells were a kind gift from Dr Helen Mathews (School of Biosciences, The University of Sheffield, UK); and MDA-MB-231 cells were a kind gift from Dr Elena Rainero (School of Biosciences, The University of Sheffield, UK). HAP1 cells (Horizon Discovery) were maintained in Iscove's modified Dulbecco's medium (IMDM, Thermo Fisher Scientific) supplemented with 10% FBS and 100 U/ml penicillin-streptomycin. Cells were maintained at 37°C and 5% CO_2_. All experiments were performed on glass coated with fibronectin (Gibco) following the manufacturer's instruction.

Transfections were performed via electroporation using the Neon system (Invitrogen) according to manufacturer's instructions using∼3μg of plasmid per transfection. Transfection was performed 18 h prior to the start of experiments. Small interfering RNA (siRNA)-mediated knockdown of genes of interest were performed using transfection via Oligofectamine (Life Technologies) according to the manufacturer's instruction. Transfection was performed 72 h prior to the start of experiments. Knockdowns were performed using RNAs previously described in Davidson et al. (2021), purchased from Qiagen. Where possible knockdown efficiency was quantified by western blot analysis.

Drug treatments were added 90 min prior to fixation and preparation for imaging. Unless otherwise stated, live-cell imaging of cells responding to treatment was performed at least 1 h after the addition of a drug. Imaging of the acute addition of a drug was performed by careful addition to the microscopy dish. All small-molecule inhibitors were used at a final concentration of 10 µM except for IPA3, which was used at 50 µM. All small-molecule inhibitors were acquired from Tocris.

For immunofluorescence experiments, cells were fixed in 4% PFA before permeabilisation in PBS with 0.1% tween 20. Blocking was performed in PBS with 5% BSA for 1 h and primary antibodies (see below) applied overnight at 4°C, in PBS with 5% BSA. Proteins were visualised by addition of fluorescent secondary antibodies (Invitrogen) and the actin cytoskeleton visualised by addition of Texas Red–phalloidin (Life Tech).

### Plasmids

Most PAK and βPIX constructs were used previously in Davidson et al., (2021). PAK PBD (amino acids 71–113) was subcloned from FL Em-PAK1 as a template. FL GFP-PAK1 (H83/86L K298R) was made via site directed mutagenesis using FL Em-PAK1 (H83/86L) as a template. GFP PAK2 ΔKin was subcloned from Addgene plasmid #23655. All other plasmids were acquired from Addgene: mApple LifeAct (Addgene plasmid #54747), mEGFP LifeAct (Addgene plasmid #54610), mCherry N-WASp (Addgene plasmid #55164), Myo1e mCherry (Addgene plasmid #27698), Emerald-Vinculin (Addgene plasmid #54303), mCherry Arp2 (Addgene plasmid #54980), GFP BTK PH (Addgene plasmid #51463), mCherry PH domain of PLCdelta1 (Addgene plasmid #36075), GFP-PH-Tapp1 (Addgene plasmid #161985), p40PX-EYFP (Addgene plasmid #19011), Cyfip GFP (Addgene plasmid #109139).

### Antibodies

Primary antibodies used in this study were: rabbit anti-human cortactin (1:1000; GTX GTX113681); mouse anti-PAK1/2/3 (1:1000; Santa Cruz Biotechnology sc-166887), rabbit anti-human GIT1 (1:1000; Cell Signalling #2919), rabbit anti P-PAK1,2,3^Ser144/141^ (1:1000; Abcam 3H12), mouse anti-β-PIX (1:1000; Santa Cruz Biotechnology sc-393184), rabbit anti-vinculin (1:400; Cell Signalling #4650), Alexa Fluor^®^ 488-conjugated anti-human CD29 antibody (1:300; BioLegend #303016), rabbit anti-AKT (1:1000; Cell Signaling #9272) and mouse anti-pAKT Ser473 (1:1000; Cell Signaling #4051).

### Microscopy

Live-cell microscopy was performed at 37°C in CO_2_ independent media without phenol red (Leibovitz L15 medium without phenol red; Thermo Fisher Scientific). Fluorescence microscopy was performed on an Olympus IX83 inverted microscope equipped with an Olympus IX3-SSU automated *xy* stage and IX3 Z-Drift compensator. Images were taken using CellSens software and recorded on a Hamamatsu imageEM x2 EM-CCD camera. TIRFm was performed with a Uapon 100×/1.49NA objective. Widefield fluorescence microscopy of live and fixed samples was performed on the same system with either a 100× or 60× objective. Images of fixed MiaPaCa cells were acquired on Nikon W1 spinning disk with a 100× objective and images of MDA-231 cells acquired with a Zeiss LSM980 Airyscan 2 with a 63× oil objective both within the Wolfson Light Microscopy facility at the University of Sheffield.

### Image analysis

All image analysis was performed in Fiji (Schindelin et al., 2019). To calculate the percentage of the perimeter of the cell marked by cortactin staining, first the total perimeter of the cell was acquired by manual segmentation. Next the length of each region of peripheral cortactin staining was measured. The cumulative total used to calculate the total percentage of cortactin staining. The number of protrusions per cell was given by the number of unique regions of peripheral cortactin staining per cell. Protrusion size was given by the length of all individual regions. The percentage of cells displaying cortactin/actin rings was acquired by counting. A cortactin/actin ring was defined as either a complete circle of dense cortactin/actin staining within the body of the cell or an incomplete circle against the periphery of the cell, behind a protrusion (marked by a narrower band of cortactin/actin staining). Examples of both are visible in Fig. 1E. The researcher doing this analysis was aware of the experimental conditions.

### *In vitro* pulldowns with lipid-coated beads

Pulldowns with lipid coated beads were performed as described previously (Hume et al., 2014). Briefly, silica microspheres were coated with phosphatidylcholine (PC) and phosphatidylserine (PS) at a molar ratio of 80:20 (Avanti Polar Lipids). These microspheres were incubated with prenylated Rac QL for 2 h before washing. This process is described in detail in Hume et al. (2014). Beads were then incubated in cell-free porcine brain extract for 30 min before extensive washing. As indicated, G5555 (10 μM) or equivalent volume of DMSO alone was added to extract immediately prior to addition to beads. Recruited proteins were detected by western blot analysis. Blots were imaged on a LI-COR Odyssey Fc imaging system and band intensities quantified using Image Studio™ software (LI-COR). Relative protein levels were quantified using Image Studio™ software. Where multiple bands were present at a relevant molecular mass (as for βPix) all bands were included in the analysis.

### Macropinocytosis

At 24 h prior to analysis, cells were seeded at a density of 1.5×10^5^ cells in 200 μl/well of complete medium into flat-bottom, 96-well plates (Thermo Fisher Scientific, 168055); each condition was seeded in duplicate, and incubated at 37°C. On the day of analysis, cells were pre-incubated with the respective inhibitors for 30 min, after which the drugs were co-incubated with 0.25 mg/ml lysine fixable Tetramethylrhodamine (TMR)-conjugated 70 kDa dextran (Thermo Fisher Scientific, D1818) for 90 min at 37°C. After incubation, cells were washed three times with cold PBS, 100 μl of trypsin (Thermo Fisher Scientific) was added to detach cells, followed by 100 μl of complete medium to neutralise the trypsin. Cells were in fixed in 50 µl/well 4% paraformaldehyde (PFA; Thermo Fisher Scientific) to a final concentration of 1%. Cells were analysed by flow cytometry using the YL1 laser on an Attune NxT Flow Cytometer (Thermo Fisher Scientific) using the high-throughput sampling attachment, which pipetted them up and down twice, before analysing 150 μl per sample at 100 μl/s. At least 10,000 cells were counted per sample. Data was then inputted to GraphPad prism and the mean of three biological replicates plotted.

### Chemotaxis

Chemotaxis assays were performed in Ibidi µ-Slide Chemotaxis chambers according to manufacturer's instructions. Chemotaxis was observed by transmitted light microscopy with a 10× objective on a Nikon ix83 inverted microscope at 37°C and cells were imaged every 5 min for 16.5 h. Cells were manually tracked using the manual tracking plugin in Fiji software. End point directionality, speed and direction autocorrelation was calculated in Microsoft Excel using DiPer as described in Gorelik and Gautreau (2014). Plots demonstrating individual tracts were also generated in DiPer.

### Western blotting

For whole-cell lysates, cells were collected in RIPA buffer [50 mM Tris-HCl pH 7.4, 150 mM NaCl, 1 mM EDTA, 10 mM NaF, 0.5% Sodium deoxycholate, 0.1% SDS, 1 mM PMSF, 1× cOmplete EDTA free protease inhibitor (Roche)]. Cells were lysed on ice with occasional vortexing. Protein concentration was determined using a Bradford assay. Samples were mixed with 4× SDS sample buffer, normalising for protein concentration and boiled for 10 min. For blotting from bead assays, beads were resuspended directly in 1× SDS sample buffer before boiling for 10 min. Samples were run on a 10% polyacrylamide gel before transferring to PVDF membrane (Invitrogen) using an iBlot 2 dry transfer system (Invitrogen). Membranes were blocked in TBS containing 5% BSA before immunoblotting overnight at 4°C overnight with indicated antibodies in TBS containing 5% BSA. Membranes were washed three times with TBS containing 0.2% Tween-20 (TBST) before incubating with secondary antibodies in TBS plus 5% BSA for 1 h. Membranes were then washed three times in TBST and imaged on a LI-COR Odyssey Fc imaging system. Uncropped images of western blots in this paper can be seen in Fig. S6.

## Supplementary Material



10.1242/joces.263763_sup1 Supplementary information

## References

[JCS263763C1] Arias-Romero, L. E. and Chernoff, J. (2008). A tale of two paks. *Biol. Cell* 100, 97-108. 10.1042/BC20070109 18199048

[JCS263763C2] Barger, S. R., Reilly, N. S., Shutova, M. S., Li, Q., Maiuri, P., Heddleston, J. M., Mooseker, M. S., Flavell, R. A., Svitkina, T., Oakes, P. W. et al. (2019). Membrane-cytoskeletal crosstalk mediated by myosin-i regulates adhesion turnover during phagocytosis. *Nat. Commun.* 10, 1249. 10.1038/s41467-019-09104-1 30890704 PMC6425032

[JCS263763C3] Baskaran, Y., Ng, Y.-W., Selamat, W., Ling, F. T. P. and Manser, E. (2012). Group i and II mammalian PAKs have different modes of activation by Cdc42. *EMBO Rep.* 13, 653-659. 10.1038/embor.2012.75 22653441 PMC3388789

[JCS263763C4] Bernitt, E., Koh, C. G., Gov, N. and Döbereiner, H.-G. (2015). Dynamics of actin waves on patterned substrates: a quantitative analysis of circular dorsal ruffles. *PLoS ONE* 10, e0115857. 10.1371/journal.pone.0115857 25574668 PMC4289068

[JCS263763C6] Bretschneider, T., Anderson, K., Ecke, M., Müller-Taubenberger, A., Schroth-Diez, B., Ishikawa-Ankerhold, H. C. and Gerisch, G. (2009). The three-dimensional dynamics of actin waves, a model of cytoskeletal self-organization. *Biophys. J.* 96, 2888-2900. 10.1016/j.bpj.2008.12.3942 19348770 PMC3325131

[JCS263763C7] Brown, J. L., Stowers, L., Baer, M., Trejo, J., Coughlin, S. and Chant, J. (1996). Human Ste20 homologue HPAK1 links GTPases to the JNK MAP kinase pathway. *Curr. Biol.* 6, 598-605. 10.1016/S0960-9822(02)00546-8 8805275

[JCS263763C8] Buracco, S., Döring, H., Engelbart, S., Singh, S. P., Paschke, P., Whitelaw, J., Thomason, P. A., Paul, N. R., Tweedy, L., Lilla, S. et al. (2024). Scar/WAVE drives actin protrusions independently of its VCA domain using proline-rich domains. *Curr. Biol.* 34, 4436-4451.e9. 10.1016/j.cub.2024.08.013 39332399

[JCS263763C9] Chaudhary, A., King, W. G., Mattaliano, M. D., Frost, J. A., Diaz, B., Morrison, D. K., Cobb, M. H., Marshall, M. S. and Brugge, J. S. (1999). Phosphatidylinositol 3-kinase regulates Raf1 through Pak phosphorylation of serine 338. *Curr. Biol.* 10, 551-554. 10.1016/S0960-9822(00)00475-9 10801448

[JCS263763C10] Chong, C., Tan, L., Lim, L. and Manser, E. (2001). The mechanism of PAK activation. *J. Biol. Chem.* 276, 17347-17353. 10.1074/jbc.M009316200 11278486

[JCS263763C11] Chow, H.-Y., Karchugina, S., Groendyke, B. J., Toenjes, S., Donovan, K. A., Fischer, E. S., Abalakov, G., Faezov, B., Dunbrack, R., Gray, N. S. et al. (2023). Development and utility of a PAK1-selective degrader. *J. Med. Chem.* 65, 15627-15641. 10.1021/acs.jmedchem.2c00756 PMC10029980 36416208

[JCS263763C12] Davidson, A., Tyler, J., Hume, P., Singh, V. and Koronakis, V. (2021). A kinase-independent function of PAK is crucial for pathogen-mediated actin Remodelling. *PLoS Pathog.* 17, e1009902. 10.1371/journal.ppat.1009902 34460869 PMC8432889

[JCS263763C13] Deacon, S. W., Beeser, A., Fukui, J. A., Rennefahrt, U. E. E., Myers, C., Chernoff, J. and Peterson, J. R. (2008). An isoform-selective, small-molecule inhibitor targets the autoregulatory mechanism of P21–activated kinase. *Chem. Biol.* 15, 322-331. 10.1016/j.chembiol.2008.03.005 18420139 PMC4353635

[JCS263763C14] Devreotes, P. N., Bhattacharya, S., Edwards, M., Iglesias, P. A., Lampert, T. and Miao, Y. (2017). Excitable signal transduction networks in directed cell migration. *Annu. Rev. Cell Dev. Biol.* 33, 103-125. 10.1146/annurev-cellbio-100616-060739 28793794 PMC5792054

[JCS263763C15] Dharmawardhane, S., Schürmann, A., Sells, M. A., Chernoff, J., Schmid, S. L. and Bokoch, G. M. (2000). Regulation of macropinocytosis by P21-activated kinase-1. *Mol. Biol. Cell* 11, 3341-3352. 10.1091/mbc.11.10.3341 11029040 PMC14996

[JCS263763C16] Edwards, D. C., Sanders, L. C., Bokoch, G. M. and Gill, G. N. (1999). Activation of LIM-kinase by Pak1 couples Rac/Cdc42 GTPase signalling to actin cytoskeletal dynamics. *Nat. Cell Biol.* 1, 253-259. 10.1038/12963 10559936

[JCS263763C17] Frost, J. A., Khokhlatchev, A., Stippec, S., White, M. A. and Cobb, M. H. (1998). Differential effects of PAK1-activating mutations reveal activity-dependent and -independent effects on cytoskeletal regulation. *J. Biol. Chem.* 273, 28191-28198. 10.1074/jbc.273.43.28191 9774440

[JCS263763C18] Gerhardt, M., Ecke, M., Walz, M., Stengl, A., Beta, C. and Gerisch, G. (2014). Actin and PIP3 waves in giant cells reveal the inherent length scale of an excited state. *J. Cell Sci.* 127, 4507-4517. 10.1242/jcs.156000 25107368

[JCS263763C19] Gorelik, R. and Gautreau, A. (2014). Quantitative and unbiased analysis of directional persistence in cell migration. *Nat. Protoc.* 9, 1931-1943. 10.1038/nprot.2014.131 25033209

[JCS263763C20] Hideki, Y. and Condeelis, J. (2014). Regulation of the actin cytoskeleton in cancer cell migration and invasion. *Biochim. Biophys. Acta* 1773, 642-652. 10.1016/j.bbamcr.2006.07.001 PMC4266238 16926057

[JCS263763C21] Higuchi, M., Onishi, K., Kikuchi, C. and Gotoh, Y. (2008). Scaffolding function of PAK in the PDK1 – Akt pathway. *Nat. Cell Biol.* 10, 1356-1364. 10.1038/ncb1795 18931661

[JCS263763C43] Hoppe, A. D. and Swanson, J. A. (2004). Cdc42, Rac1, and Rac2 display distinct patterns of activation during phagocytosis. *Mol. Biol. Cell* 15, 3509-3519. 10.1091/mbc.e03-11-0847 15169870 PMC491814

[JCS263763C22] Hume, P. J., Humphreys, D. and Koronakis, V. (2014). *WAVE Regulatory Complex Activation. Reconstituting the Cytoskeleton*, Vol. 540, 1st edn. Elsevier Inc. 10.1016/B978-0-12-397924-7.00020-0 24630117

[JCS263763C23] Itakura, A., Aslan, J. E., Kusanto, B. T., Phillips, K. G., Porter, J. E., Newton, P. K., Nan, X., Insall, R. H., Chernoff, J. and McCarty, O. J. T. (2013). P21-Activated kinase (PAK) regulates cytoskeletal reorganization and directional migration in human neutrophils. *PLoS ONE* 8, e73063. 10.1371/journal.pone.0073063 24019894 PMC3760889

[JCS263763C24] Kage, F., Do, H., Mietkowska, M., Schaks, M., Gru, F., Stahnke, S., Steffen, A., Müsken, M., Stradal, T. E. B. and Rottner, K. (2022). Lamellipodia-like actin networks in cells lacking WAVE regulatory complex. *J. Cell Sci.* 135, jcs260364. 10.1242/jcs.260364 35971979 PMC9511706

[JCS263763C25] Karpov, A. S., Amiri, P., Bellamacina, C., Bellance, M.-H., Breitenstein, W., Daniel, D., Denay, R., Fabbro, D., Fernandez, C., Galuba, I. et al. (2015). Optimization of a dibenzodiazepine hit to a potent and selective allosteric PAK1 inhibitor. *ACS Med. Chem. Lett.* 6, 776-781. 10.1021/acsmedchemlett.5b00102 26191365 PMC4499825

[JCS263763C26] Karunarathne, W. K. A., Giri, L., Patel, A. K., Venkatesh, K. V. and Gautam, N. (2013). Optical control demonstrates switch-like PIP3 dynamics underlying the initiation of immune cell migration. *Proc. Natl. Acad. Sci. U.S.A.* 110, E1575-E1583. 10.1073/pnas.1220755110 23569254 PMC3637758

[JCS263763C27] Kim, M., Ogawa, M., Fujita, Y., Yoshikawa, Y., Nagai, T., Koyama, T., Nagai, S., Lange, A., Fässler, R. and Sasakawa, C. (2009). Bacteria hijack integrin-linked kinase to stabilize focal adhesions and block cell detachment. *Nature* 459, 578-582. 10.1038/nature07952 19489119

[JCS263763C28] Kim, S. H., Song, J. H., Kim, M. J., Song, M. G., Ku, A. A., Bandyopadhyay, S., McCormick, F. and Kim, S. E. (2022). Novel regulators of macropinocytosis-dependent growth revealed by informer set library screening in pancreatic cancer cells. *Metabolites* 12, 831. 10.3390/metabo12090831 36144235 PMC9502772

[JCS263763C29] King, H., Nicholas, N. S. and Wells, C. M. (2014). *Role of P-21-Activated Kinases in Cancer Progression. International Review of Cell and Molecular Biology*, Vol. 309, 1st edn. Elsevier Inc. 10.1016/B978-0-12-800255-1.00007-7 24529727

[JCS263763C30] Kuhn, J., Banerjee, P., Haye, A., Robinson, D. N., Iglesias, P. A. and Devreotes, P. N. (2024). Complementary cytoskeletal feedback loops control signal transduction excitability and cell polarity. *bioRxiv*, 13:2024.02.13.580131. 10.1101/2024.02.13.580131 PMC12344061 40796751

[JCS263763C31] Kumar, R., Sanawar, R., Li, X. and Li, F. (2017). Structure, biochemistry, and biology of PAK kinases. *Gene* 605, 20-31. 10.1016/j.gene.2016.12.014 28007610 PMC5250584

[JCS263763C32] Lee, S.-W., Zhang, Y., Jung, M., Cruz, N., Alas, B. and Commisso, C. (2019). EGFR-Pak signaling selectively regulates article EGFR-Pak signaling selectively regulates. *Dev. Cell* 50, 381-392.e5. 10.1016/j.devcel.2019.05.043 31257175 PMC6684838

[JCS263763C33] Lei, M., Lu, W., Meng, W., Parrini, M.-C., Eck, M. J., Mayer, B. J. and Harrison, S. C. (2000). Structure of PAK1 in an autoinhibited conformation reveals a multistage activation switch. *Cell* 102, 387-397. 10.1016/S0092-8674(00)00043-X 10975528

[JCS263763C34] Lutton, J. E., Coker, H. L. E., Paschke, P., Munn, C. J., King, J. S., Bretschneider, T. and Kay, R. R. (2023). Formation and closure of macropinocytic cups in dictyostelium Ll Ll formation and closure of macropinocytic cups in dictyostelium. *Curr. Biol.* 33, 3083-3096.e6. 10.1016/j.cub.2023.06.017 37379843 PMC7614961

[JCS263763C35] Manser, E., Leung, T., Salihuddin, H., Zhao, Z.-s. and Lim, L. (1994). ARTICLES a brain serine/threonine protein kinase activated by Cdc42 and Racl. *Nature* 367, 40-46. 10.1038/367040a0 8107774

[JCS263763C36] Manser, E., Chong, C., Zhao, Z.-S., Leung, T., Michael, G., Hall, C. and Lim, L. (1995). Molecular cloning of a new member of the P21-Cdc42/Rac-activated kinase (PAK) family. *J. Biol. Chem.* 270, 25070-25078. 10.1074/jbc.270.42.25070 7559638

[JCS263763C37] Manser, E., Huang, H.-Y., Loo, T.-H., Chen, X.-Q., Dong, J.-M., Leung, T. and Lim, L. (1997). Expression of constitutively active Alpha-PAK reveals effects of the kinase on actin and focal complexes. *Mol. Cell. Biol.* 17, 1129-1143. 10.1128/mcb.17.3.1129 9032240 PMC231838

[JCS263763C38] Manser, E., Loo, T.-H., Koh, C.-G., Zhao, Z.-S., Chen, X.-Q., Tan, L., Tan, I., Leung, T. and Lim, L. (1998). PAK kinases are directly coupled to the PIX family of nucleotide exchange factors. *Mol. Cell* 1, 183-192. 10.1016/S1097-2765(00)80019-2 9659915

[JCS263763C78] Martinez, M. L., Nan, K., Bao, Z., Bacchetti, R., Yuan, S., Tyler, J., Guezennec, X. L., Bard, F. A. and Rainero, E. (2024). Novel kinase regulators of extracellular matrix internalisation identified by high-content screening modulate invasive carcinoma cell migration. *PLoS Biol.* 22, e3002930. 10.1371/journal.pbio.3002930 39666682 PMC11637276

[JCS263763C39] Masters, T. A., Sheetz, M. P. and Gauthier, N. C. (2016). F-Actin waves, actin cortex disassembly and focal exocytosis driven by actin-phosphoinositide positive feedback. *Cytoskeleton* 73, 180-196. 10.1002/cm.21287 26915738

[JCS263763C40] Miao, Y., Bhattacharya, S., Edwards, M., Cai, H., Inoue, T., Iglesias, P. A. and Devreotes, P. N. (2017). Altering the threshold of an excitable signal transduction network changes cell migratory modes. *Nat. Cell Biol.* 19, 329-340. 10.1038/ncb3495 28346441 PMC5394931

[JCS263763C41] Miao, Y., Bhattacharya, S., Banerjee, T., Abubaker-Sharif, B., Long, Y., Inoue, T., Iglesias, P. A. and Devreotes, P. N. (2019). Wave patterns organize cellular protrusions and control cortical dynamics. *Mol. Syst. Biol.* 15, e8585. 10.15252/msb.20188585 30858181 PMC6413885

[JCS263763C42] Mierke, C. T., Puder, S., Aermes, C., Fischer, T. and Kunschmann, T. (2020). Effect of PAK inhibition on cell mechanics depends on Rac1. *Front. Cell Dev. Biol.* 8, 13. 10.3389/fcell.2020.00013 32047750 PMC6997127

[JCS263763C44] Nazemi, M., Yanes, B., Walker, H. J., Pham, K., Collins, M. O., Bard, F. and Rainero, E. (2024). The extracellular matrix supports breast cancer cell growth under amino acid starvation by promoting tyrosine catabolism. *PLoS Biol.* 22, e3002406. 10.1371/journal.pbio.3002406 38227562 PMC10791009

[JCS263763C45] Olson, M. F. and Sahai, E. (2009). The actin cytoskeleton in cancer cell motility. *Clin. Exp. Metastasis* 26, 273-287. 10.1007/s10585-008-9174-2 18498004

[JCS263763C46] Ong, C. C., Jubb, A. M., Haverty, P. M., Zhou, W., Tran, V., Truong, T., Turley, H., O'Brien, T., Vucic, D., Harris, A. L. et al. (2011). Targeting p21-activated kinase 1 (PAK1) to induce apoptosis of tumor cells. *Proc. Natl. Acad. Sci. USA* 108, 7177-7182. 10.1073/pnas.1103350108 21482786 PMC3084065

[JCS263763C47] Ostrowski, P. P., Freeman, S. A., Fairn, G., Ostrowski, P. P., Freeman, S. A., Fairn, G. and Grinstein, S. (2019). Dynamic podosome-like structures in nascent phagosomes are coordinated by phosphoinositides article dynamic podosome-like structures in nascent phagosomes are coordinated by phosphoinositides. *Dev. Cell* 50, 397-410.e3. 10.1016/j.devcel.2019.05.028 31231039

[JCS263763C48] Pal, D. S., Banerjee, T., Lin, Y., de Trogoff, F., Borleis, J., Iglesias, P. A. and Devreotes, P. N. (2023). Actuation of single downstream nodes in growth factor network steers immune cell migration. *Dev. Cell* 58, 1170-1188.e7. 10.1016/j.devcel.2023.04.019 37220748 PMC10524337

[JCS263763C49] Papakonstanti, E. A. and Stournaras, C. (2002). Association of PI-3 kinase with PAK1 leads to actin phosphorylation and cytoskeletal reorganization. *Mol. Biol. Cell* 13, 2946-2962. 10.1091/mbc.02-01-0599 12181358 PMC117954

[JCS263763C50] Parka, E. R., Eblenb, S. T. and Catling, A. D. (2008). MEK1 activation by PAK: a novel mechanism. *Cell. Signal.* 19, 1488-1496. 10.1016/j.cellsig.2007.01.018 PMC2233889 17314031

[JCS263763C51] Premont, R. T., Perry, S. J., Schmalzigaug, R., Roseman, T. J., Xing, Y. and Claing, A. (2004). The GIT/PIX complex: an oligomeric assembly of GIT family ARF GTPase-activating proteins and PIX family Rac1/Cdc42 guanine nucleotide exchange factors. *Cell. Signal.* 16, 1001-1011. 10.1016/j.cellsig.2004.02.002 15212761

[JCS263763C5] Quinn, S. E., Huang, L., Kerkvliet, J. G., Swanson, J. A., Smith, S., Hoppe, A. D., Anderson, R. B., Thiex, N. W. and Scott, B. L. (2021). The structural dynamics of macropinosome formation and PI3–kinase-mediated sealing revealed by lattice light sheet microscopy. *Nat. Commun.* 12, 4838. 10.1038/s41467-021-25187-1 34376698 PMC8355319

[JCS263763C52] Radu, M., Semenova, G., Kosoff, R. and Chernoff, J. (2014). PAK signalling during the development. *Nat. Rev. Cancer* 14, 13-25. 10.1038/nrc3645 24505617 PMC4115244

[JCS263763C53] Sanders, L. C., Matsumura, F., Bokoch, G. M. and de Lanerolle, P. (1999). Inhibition of myosin light chain kinase by P21–activated kinase. *Science* 283, 2083-2086. 10.1126/science.283.5410.2083 10092231

[JCS263763C54] Schindelin, J., Arganda-Carreras, I., Frise, E., Kaynig, V., Longair, M., Pietzsch, T., Preibisch, S., Rueden, C., Saalfeld, S., Schmid, B. et al. (2019). Fiji: an open-source platform for biological-image analysis. *Sci. Rep.* 9, 3794. 10.1038/nmeth.2019 22743772 PMC3855844

[JCS263763C55] Sells, M. A., Knaus, U. G., Bagrodia, S., Ambrose, D. M., Bokoch, G. M. and Chernoff, J. (1997). Human P21-activated kinase (Pak1) regulates actin organization in mammalian cells. *Curr. Biol.* 7, 202-210. 10.1016/S0960-9822(97)70091-5 9395435

[JCS263763C56] Sells, M. A., Boyd, J. T. and Chernoff, J. (1999). P21-activated kinase 1 (Pak1) regulates cell motility in mammalian fibroblasts. *J. Cell Biol.* 145, 837-849. 10.1083/jcb.145.4.837 10330410 PMC2133181

[JCS263763C57] Semenova, G. and Chernoff, J. (2017). Targeting PAK1. *Biochem. Soc. Trans.* 45, 79-88. 10.1042/BST20160134 28202661 PMC5973817

[JCS263763C58] Sorrell, F. J., Kilian, L. M. and Elkins, J. M. (2019). Solution structures and biophysical analysis of full-length group A PAKs reveal they are monomeric and auto-inhibited in *Cis*. *Biochem. J.* 476, 1037-1051. 10.1042/BCJ20180867 30858169 PMC6448136

[JCS263763C59] Sugiyama, N., Imamura, H. and Ishihama, Y. (2019). Large-scale discovery of substrates of the human kinome. *Sci. Rep.* 9, 10503. 10.1038/s41598-019-46385-4 31324866 PMC6642169

[JCS263763C60] Swanson, J. A., Yoshida, S. and Swanson, J. A. (2019). Macropinosomes as units of signal transduction. *Philos. Trans. R. Soc. Lond. B Biol. Sci.* 374, 20180157. 10.1098/rstb.2018.0157 30967006 PMC6304739

[JCS263763C61] Vadlamudi, R. K., Li, F., Adam, L., Nguyen, D., Ohta, Y., Stossel, T. P. and Kumar, R. (2003). Filamin is essential in actin cytoskeletal assembly mediated by P21-activated Kinase 1. *Nat. Cell. Biol.* 4, 681-690. 10.1038/ncb838 12198493

[JCS263763C62] Vadlamudi, R. K., Li, F., Barnes, C. J., Bagheri-Yarmand, R. and Kumar, R. (2004). P41-Arc subunit of human Arp2/3 complex is a P21-activated kinase-1-interacting substrate. *EMBO Rep.* 5, 154-160. 10.1038/sj.embor.7400079 14749719 PMC1298990

[JCS263763C63] van den Dries, K., Nahidiazar, L., Slotman, J. A., Meddens, M. B. M., Pandzic, E., Joosten, B., Ansems, M., Schouwstra, J., Meijer, A., Steen, R. et al. (2019). Modular actin nano-architecture enables podosome protrusion and mechanosensing. *Nat. Commun.* 10, 5171. 10.1038/s41467-019-13123-3 31729386 PMC6858452

[JCS263763C64] Veltman, D. M., Lemieux, M. G., Knecht, D. A. and Insall, R. H. (2014). PIP 3 -Dependent macropinocytosis is incompatible with chemotaxis. *J. Cell. Biol.* 204, 497-505. 10.1083/jcb.201309081 24535823 PMC3926956

[JCS263763C65] Veltman, D. M., Williams, T. D., Bloomfield, G., Chen, B.-C., Betzig, E., Insall, R. H. and Kay, R. R. (2016). A plasma membrane template for macropinocytic cups. *Elife* 5, e20085. 10.7554/eLife.20085 27960076 PMC5154761

[JCS263763C66] Vicker, M. G. (2002a). Eukaryotic cell locomotion depends on the propagation of self-organized reaction-diffusion waves and oscillations of actin filament assembly. *Exp. Cell Res.* 275, 54-66. 10.1006/excr.2001.5466 11925105

[JCS263763C67] Vicker, M. G. (2002b). F-Actin assembly in dictyostelium cell locomotion and shape oscillations propagates as a self-organized reaction-diffusion wave. *FEBS Lett.* 510, 7-11. 10.1016/S0014-5793(01)03207-0 11755520

[JCS263763C68] Weed, S. A., Karginov, A. V., Schafer, D. A., Weaver, A. M., Kinley, A. W., Cooper, J. A. and Thomas Parsons, J. (1999). Cortactin localization to sites of actin assembly in lamellipodia requires interactions with F-Actin and the Arp2/3 complex. *J. Cell Biol.* 151, 29-40. 10.1083/jcb.151.1.29 PMC2189811 11018051

[JCS263763C69] Xiao, G.-H., Beeser, A., Chernoff, J. and Testa, J. R. (2002). P21-activated kinase links Rac/Cdc42 signaling to merlin. *J. Biol. Chem.* 277, 883-886. 10.1074/jbc.C100553200 11719502

[JCS263763C70] Ye, D. Z. and Field, J. (2012). PAK signaling in cancer. *Cell Logist.* 2, 105-116. 10.4161/cl.21882 23162742 PMC3490961

[JCS263763C71] Yin, G., Zheng, Q., Yan, C. and Berk, B. C. (2005). GIT1 is a scaffold for ERK1/2 activation in focal adhesions. *J. Biol. Chem.* 280, 27705-27712. 10.1074/jbc.M502271200 15923189

[JCS263763C72] Zang, M., Hayne, C. and Luo, Z. (2002). Interaction between active Pak1 and Raf-1 is necessary for phosphorylation and activation of Raf-1. *J. Biol. Chem.* 277, 4395-4405. 10.1074/jbc.M110000200 11733498

[JCS263763C73] Zhan, H., Bhattacharya, S., Cai, H., Iglesias, P. A., Huang, C.-H., Devreotes, P. N. and Zhan, H. (2020). An excitable Ras/PI3K/ERK signaling network controls migration and oncogenic transformation in Ll article an excitable Ras/PI3K/ERK signaling network controls migration and oncogenic transformation in epithelial cells. *Dev. Cell* 54, 608-623.e5. 10.1016/j.devcel.2020.08.001 32877650 PMC7505206

[JCS263763C74] Zhan, H., Pal, D. S., Borleis, J., Janetopoulos, C., Huang, C.-H. and Peter, N. (2024). Self-organizing glycolytic waves fuel cell migration and cancer progression. *bioRxiv*, 28:2024.01.28.577603. 10.1101/2024.01.28.577603

[JCS263763C75] Zhao, Z.-S., Manser, E., Chen, X.-Q., Chong, C., Leung, T. and Lim, L. (1998). A conserved negative regulatory region in PAK: inhibition of PAK kinases reveals their morphological roles downstream of Cdc42 and Rac1. *Mol. Cell Biol.* 18, 2153-2163. 10.1128/MCB.18.4.2153 9528787 PMC121452

[JCS263763C76] Zhu, G., Wang, Y., Huang, B., Liang, J., Ding, Y., Xu, A. and Wu, W. (2012). A Rac1/PAK1 cascade controls b -catenin activation in colon cancer cells. *Oncogene* 31, 1001-1012. 10.1038/onc.2011.294 21822311

[JCS263763C77] Zhu, J., Zhou, Q., Xia, Y., Lin, L., Li, J., Peng, M. and Zhang, R. (2020). Article GIT/PIX condensates are modular and ideal for distinct compartmentalized cell signaling Ll Article GIT/PIX condensates are modular and ideal for distinct compartmentalized cell signaling. *Mol. Cell* 79, 782-796.e6. 10.1016/j.molcel.2020.07.004 32780989

