# Supplementary material for: A role for class I p21-activated kinases in the regulation of the excitability of the actin cytoskeleton
Source: J Cell Sci. 2025 Jun 23;138(12):jcs263763. doi: 10.1242/jcs.263763 (PMC12273633; doi:10.1242/jcs.263763)
Supplement: Supplementary information [file joces-138-263763-s1.pdf]

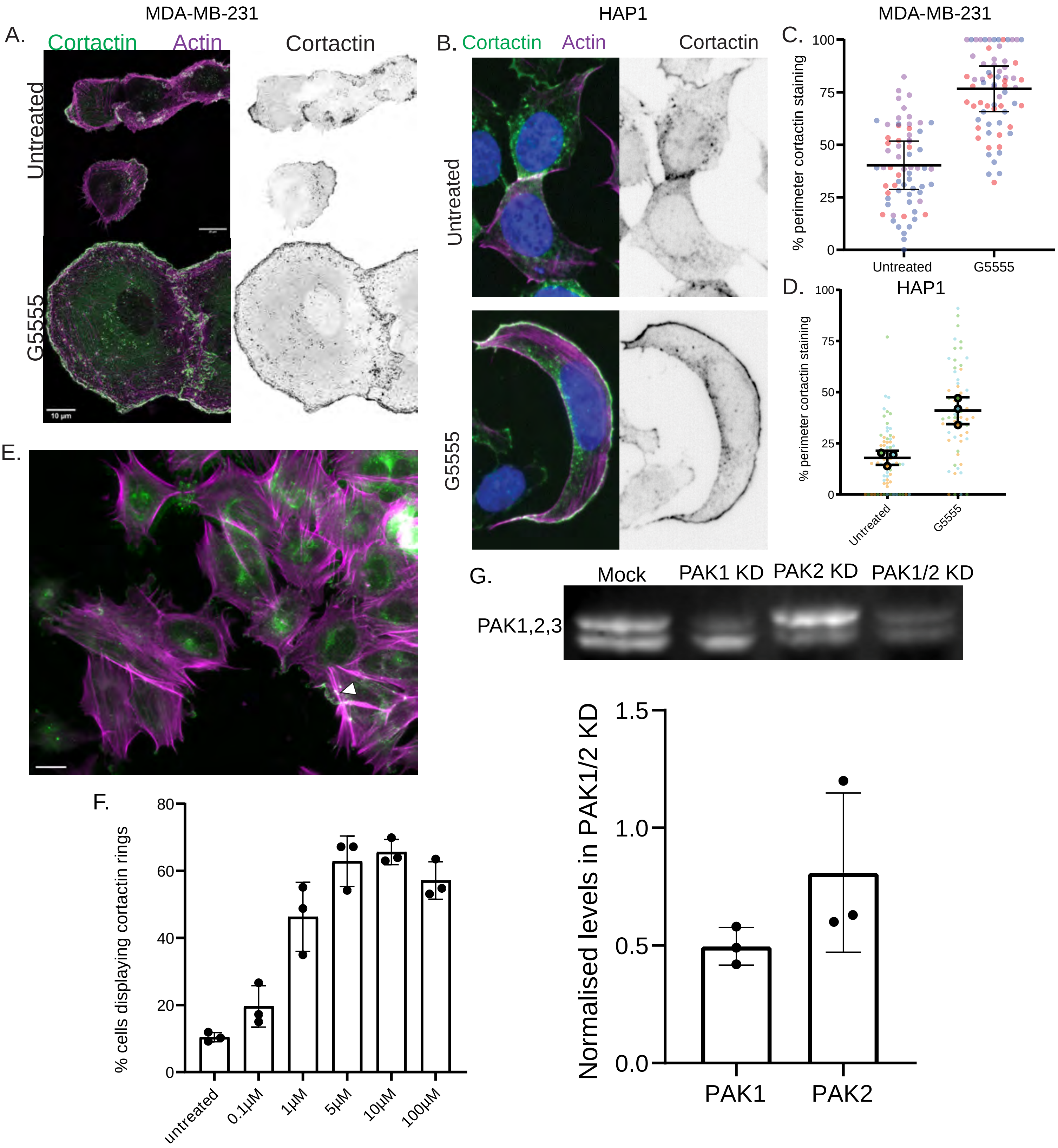

Fig. S1. Class 1 PAK kinase inhibition with small molecule inhibitor A. Representative images of MDA-MB-231 cells highlighting increase in perimeter cortactin staining following addition of 10µM G5555 for 1 hour B. Representative images of HAP1 cells highlighting increase in perimeter cortactin staining follow-ing addition of 10µM G5555 for 1 hour. C. Quantification of the percentage of perimeter of cell marked by cortactin in MDA-MB-231 cells with or without G5555. D. Quantification of the percentage of perimeter of cell marked by cortactin in HAP1 cells with or without G5555. E. Representatitive image of MEFs treated with 1:1000 µl DMSO. arrowhead indicates cortactin ring. F. Percentage of MEFs displaying cortactin rings one hour after the addition of indicated concentration of G5555 G. Blot indicating loss of bands observed with PAK1,2,3 antibody following indicated treatment. accompanying graph provides quantification of knockdown efficiency for experiments included in figure 1G. Pak levels normalised to β actin loading control and PAK levels in mock treated extracts.

Journal of Cell Science • Supplementary information

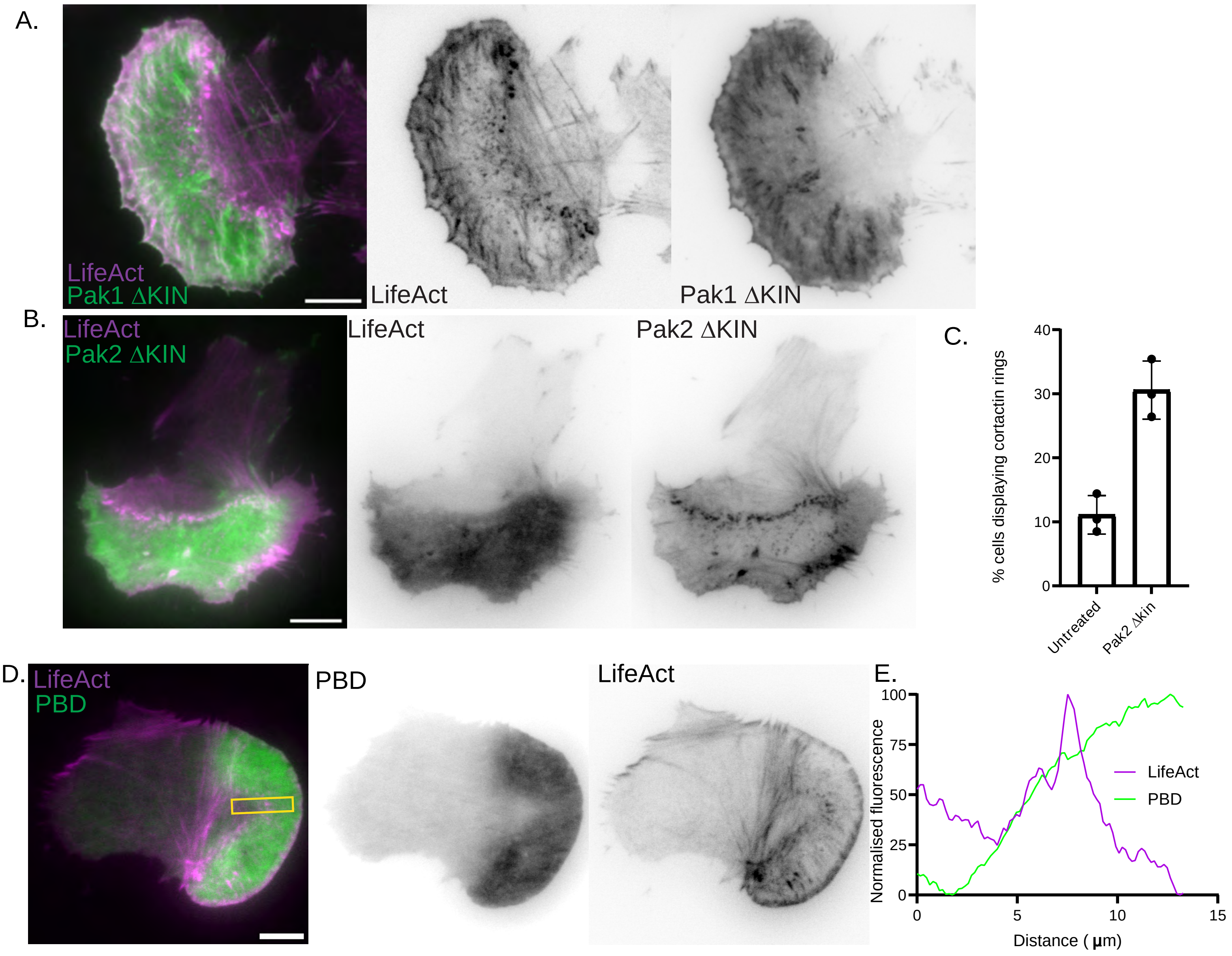

Fig. S2. TIRFm of Truncated PAK1 and PAK2 constructs. A. TIRFm image of a MEF overex-pressing GFP-PAK1-ΔKIN and mcherry LifeAct. See large fan-shaped lammellipodia. B. TIRFm image of a MEF overexpressing GFP-PAK2-ΔKIN and mcherry LifeAct. Again, large fan shaped lammellipodia present. C. Quantification of percentage of cells expressing GFP-PAK2-ΔKIN that form cortactin rings. Quantification performed on cells fixed and stained for cortactin and imaged by widefield microscopy D. Although GFP-PAK PBD is insufficient to drive the formation of cortactin rings, it is localised to these structures following addition of G5555. Representative TIRFm image of MEF expressing GFP-PAK PBD and LifeAct following treatment with µM G5555 for atleast an hour. E. Line scan taken along position indicated in Supplemental Figure 2D highlighting localisation of GFP-PAK PBD relative to peak of LifeAct signal that represents the edge of the cortactin/ actin ring structure. All scale bars 10µm.

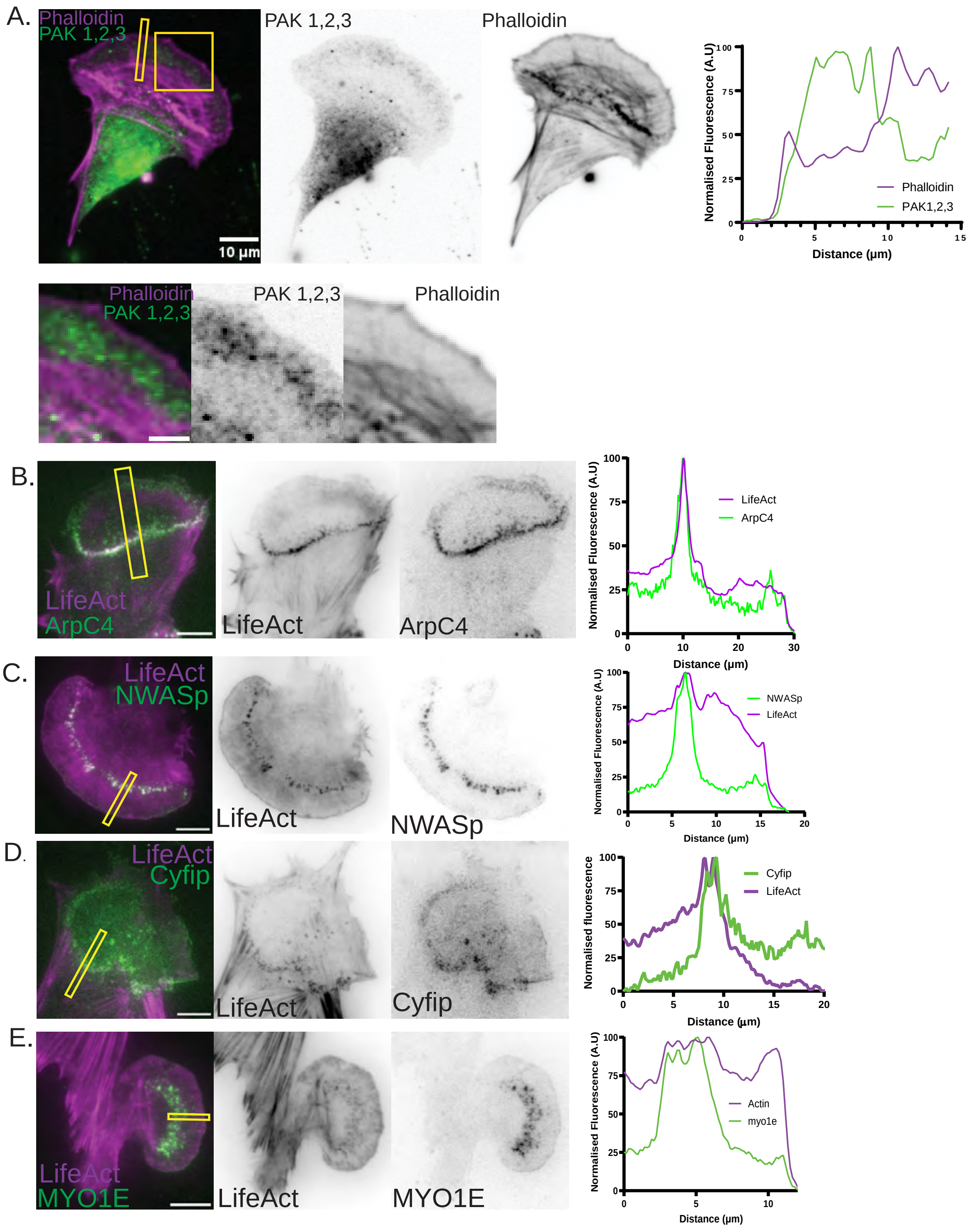

Fig. S3. Localisation of endogenous class I PAKs and actin regulators relative to actin ring. A. Widefield fluoresence microscopy image of MEF fixed and stained for endogenous class I PAKs and actin following treatment with 10 µM G5555 for 1 hour. Accompanying linescan taken along rectangle indicated in two colour image. Inset taken from indicated square box and highlights PAK1,2,3 signal within actin ring. inset scale bar 5 µm. B-E. Representative TIRFm image of cell expressing LifeAct (shown in magenta) and indicat-ed fluorescently tagged actin regulator (shown in green), treated with 10 µM G5555 for atleast an hour. Accompanying linescan highlights localisation of regulator to actin ring. A. MEF expressing mcherry-ArpC4 and mEGFP-LifeAct. B. MEF expressing mCherry-N-WASp and mEGFP-LifeAct. C. MEF expressing Cyfip-GFP and mCherry-LifeAct. Scale bar in magnified panel 5µm. D. MEF expressing Myo1e-mCherry and mEGFP LifeAct. All scale bars 10µm unless stated.

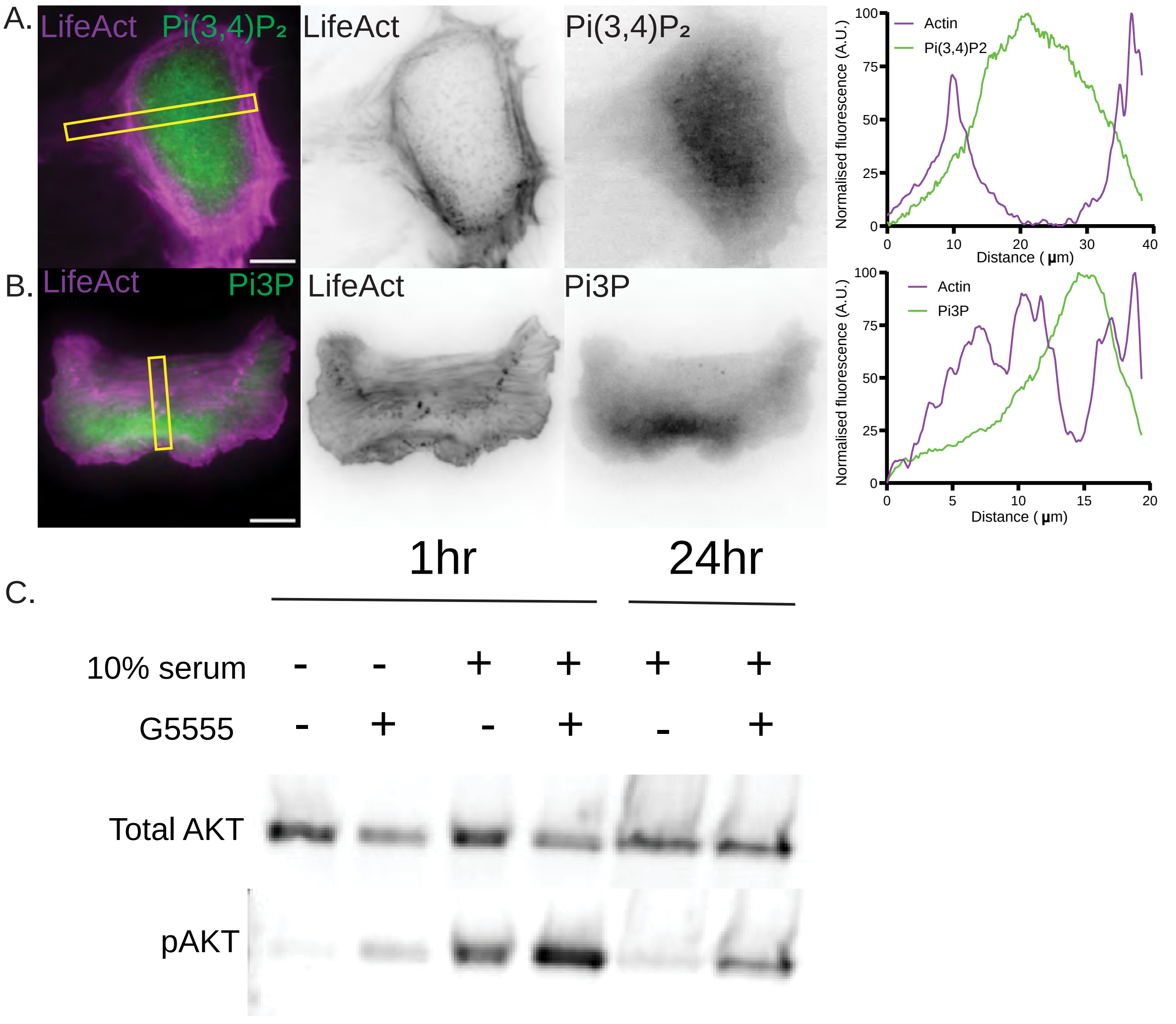

Fig. S4. Localisation of 3' phosphoinositide's and change in pAKT signal-ling. A. TIRFm image of MEF transiently overexpressing mCherry-LifeAct and Pi(3,4)P_2 marker (GFP PH TAPP1) following treatment with 10 μM G5555 for at least an hour. Accompanying linescan demonstrates localization Pi(3,4)P_2 marker relative to actin cytoskeleton. B. TIRFm image of MEF transiently overexpressing mCherry-LifeAct and Pi3P marker (GFP p40PX-EYFP) following treatment with 10 μM G5555 for at least an hour. Accompanying linescan demonstrates localization Pi3P marker relative to actin cytoskeleton. C. Western blot showing change in pAKT levels in response to addition of 10 μM G5555 for the indicated time in the presence or absense of 10% serum. All scale bars 10μm.

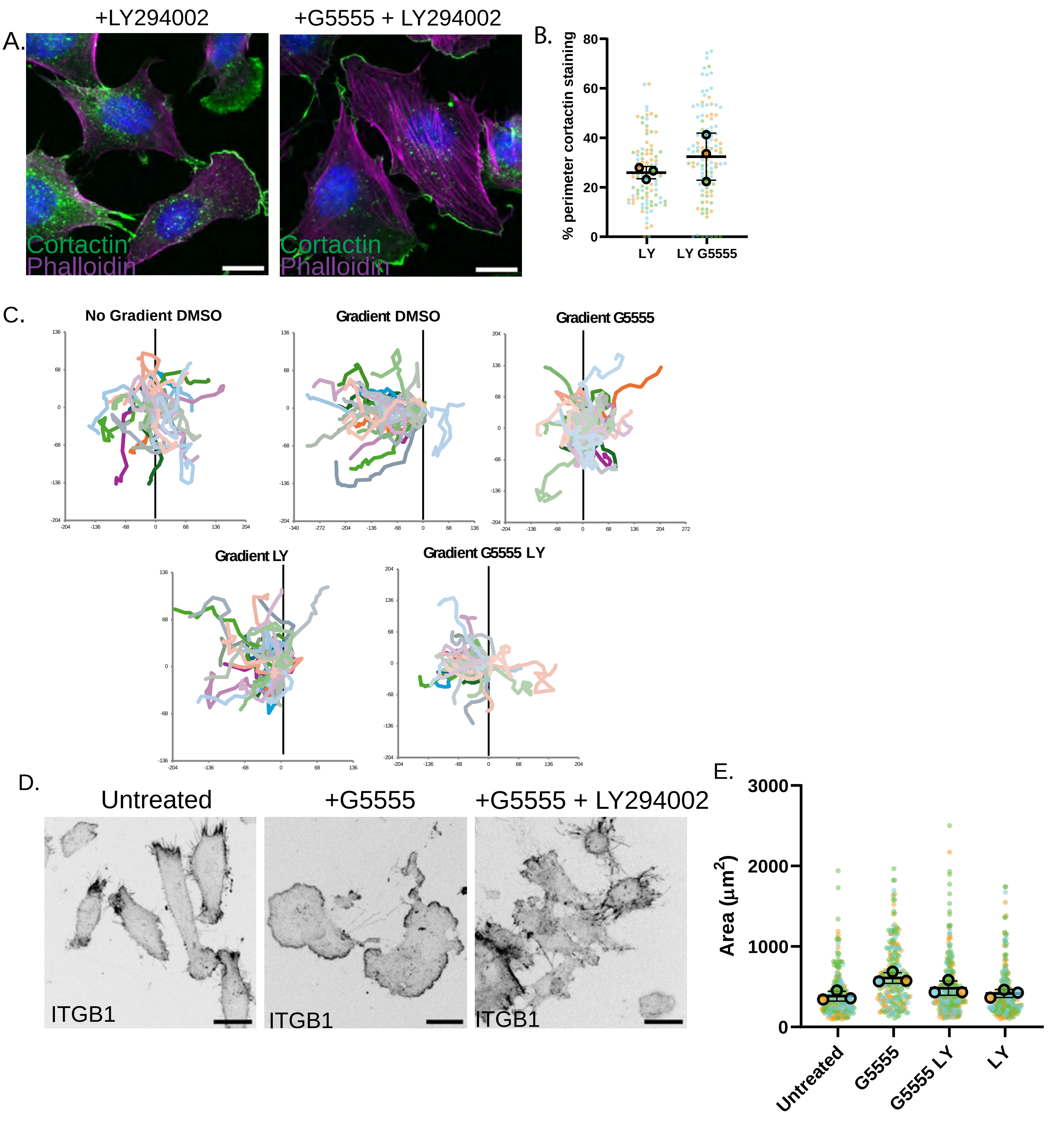

Fig. S5. PI3K dependence of phenotypes observed upon PAK kinase inhibition. A. Representative images showing cortactin localization in cells treated with LY294002 or cotreated with LY294002 and G5555 B. Quantification of the percentage of perimeter of cell marked by cortac-tin in cells treated with LY294002 or cotreated with LY294002 and G5555. C. Subset of individual tracks from chemotaxis assay (Figure 5B). Cells migrating in indicated condition. Gradient indicates presence of a chemotactic gradient. Tracks represent one biological repeat and are presented here to highlight the effect of inhibitors on chemotaxis D. Representative images of MiaPacca cells in indicated conditions. Cells were fixed and stained for ITGB1 to allow visualisation. E. Quantification of MiaPacca cell area in indicated conditions. All inhibitors used at 10 μM. Inhibitors added one hour before fixation. In the case of migration data, tracking was initiated 1 hour after the start of the assay, to allow cells to respond to inhibitors. All scale bars 10µm.

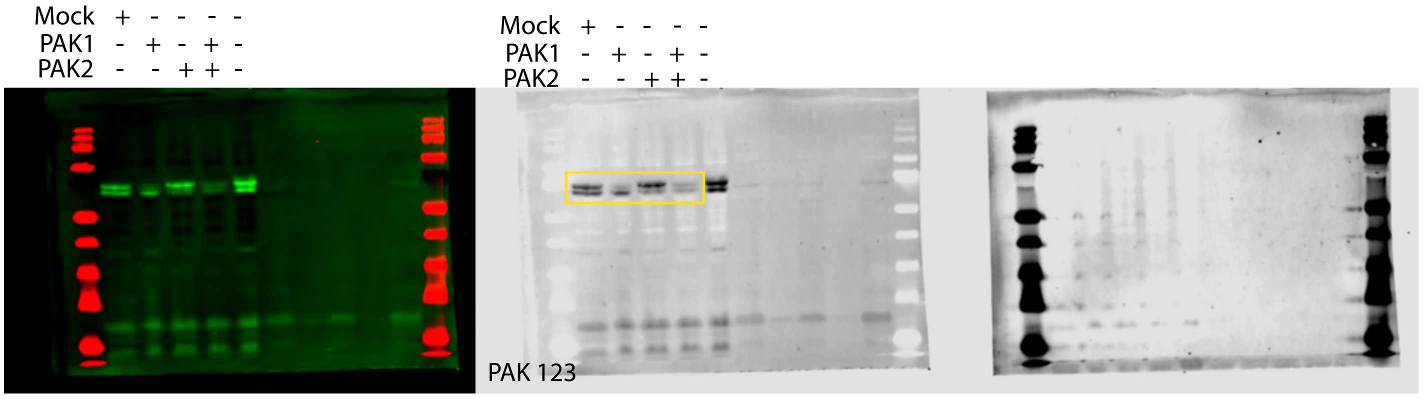

Blot used in supp. figure 1F. Demonstrates loss of bands following PAK1, PAK2, and PAK1/2 KD. Was used to determine how to quantify KD efficiency. Box indicates region used in the figure. Antibodies used indicated in the bottom corner of the blot. Other channel included to show ladder.

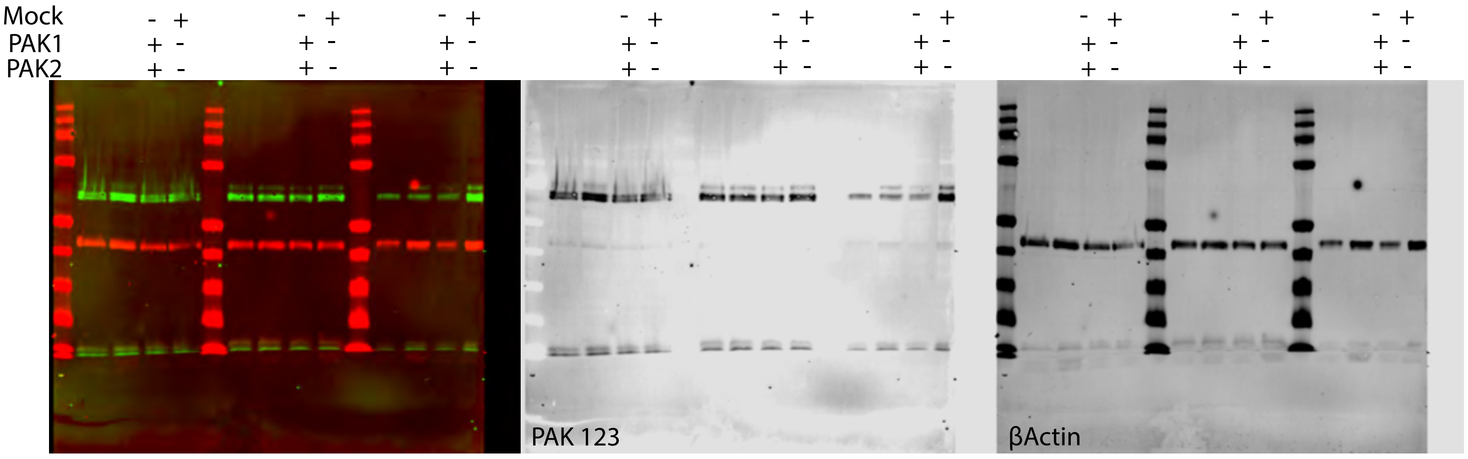

Blot used to determine KD efficiency reported in supp. figure 1F. Antibodies used indicated in the bottom corner of the blot.

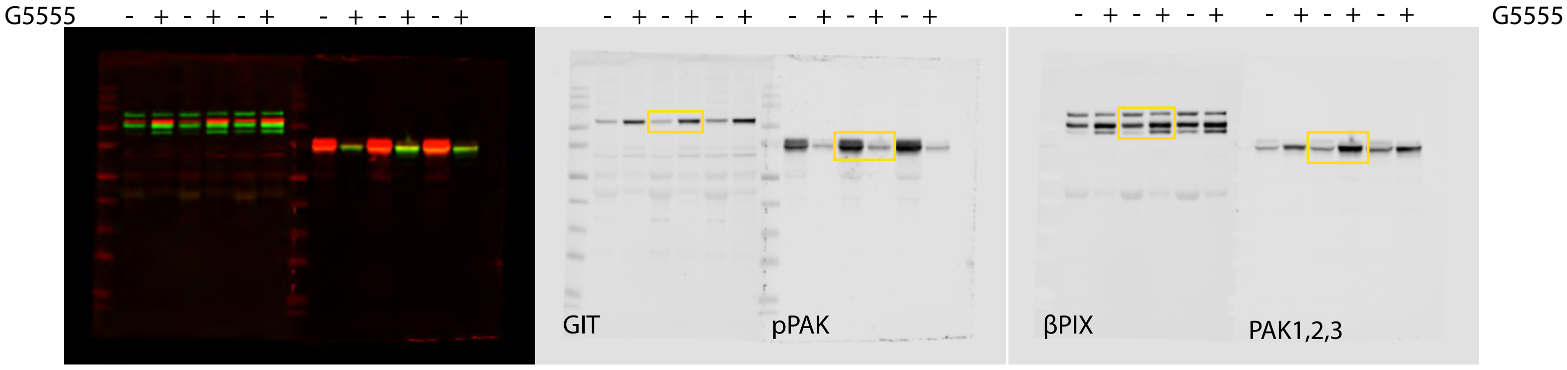

Blot that makes up the majority of fig2D. Boxes indicate regions used in the figure. Antibodies used indicated in the bottom corner of the blot.

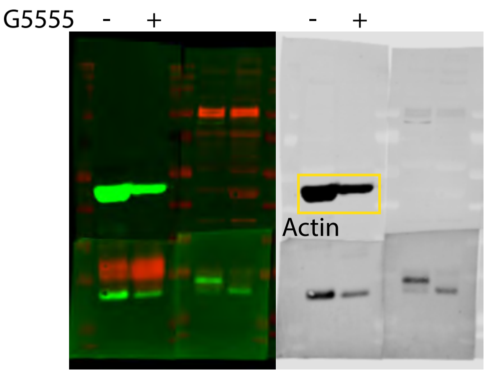

Blot that makes up the rest of fig 2D. Box indicate region used in the figure.Antibodies used indicated in the bottom corner of the blot.

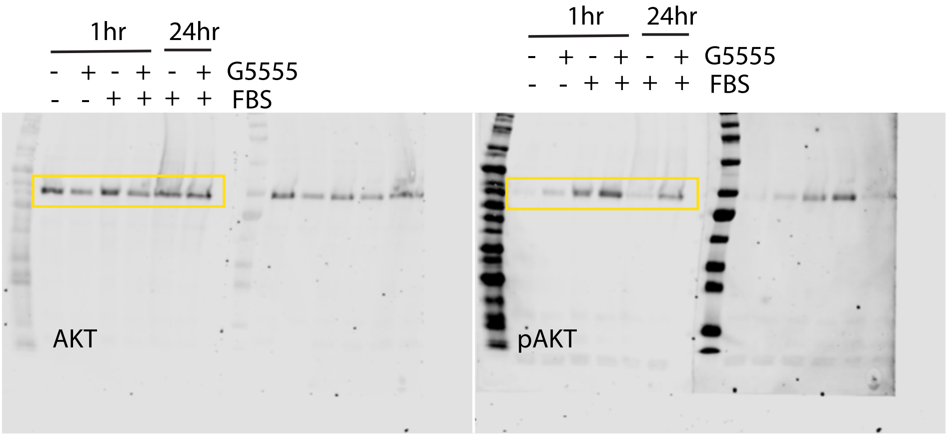

Blot that makes up supp. figure 4C. Boxes indicate regions used in the figure. Antibodies used indicated in the bottom corner of the blot.

Fig. S6. Blot Transparency

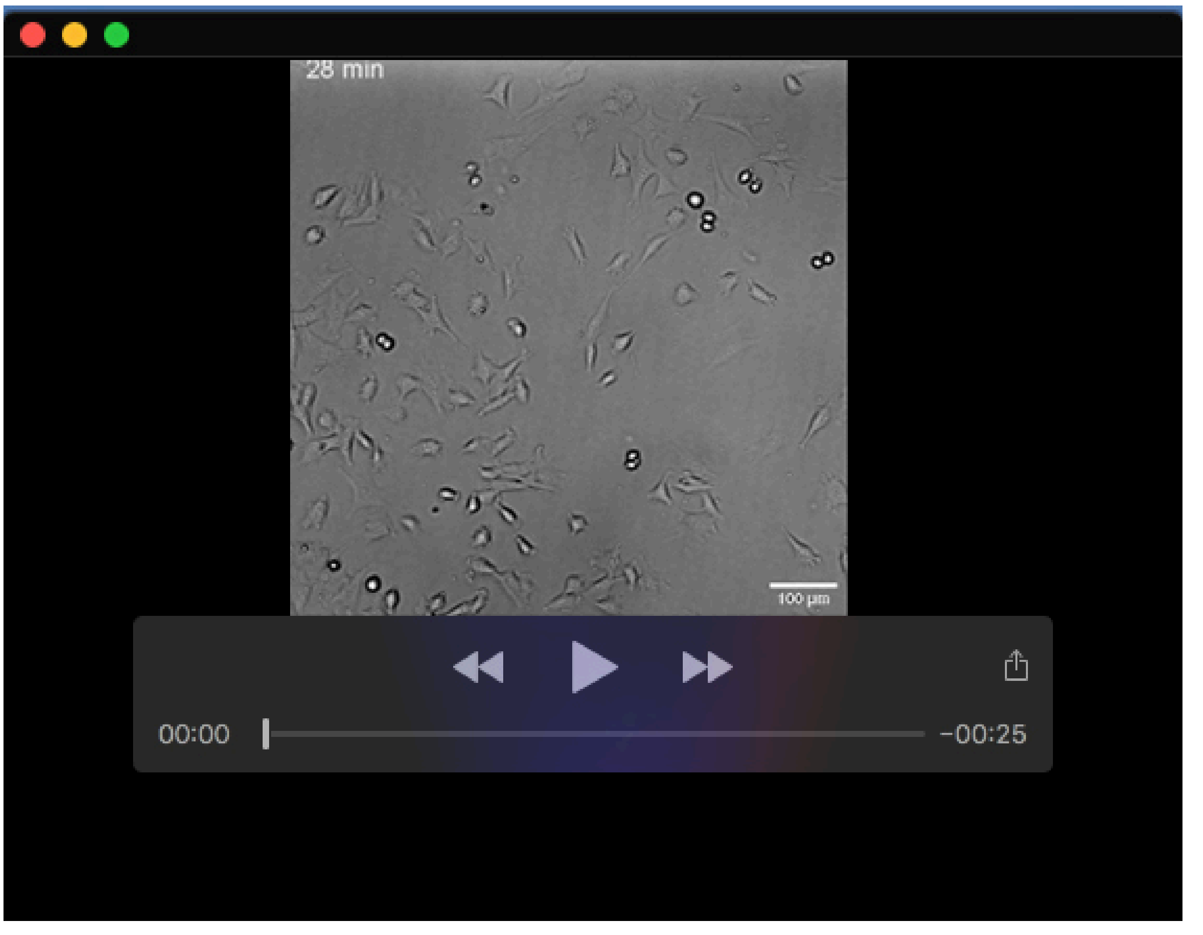

Movie 1. MEFs rapidly polarise following the addition of class I PAK kinase inhibitor. Low magnification movie of MEFs responding to the addition of 10 µM G5555. Addition of the drug occurs after 42 minutes, and the point of addition is indicated in the movie.

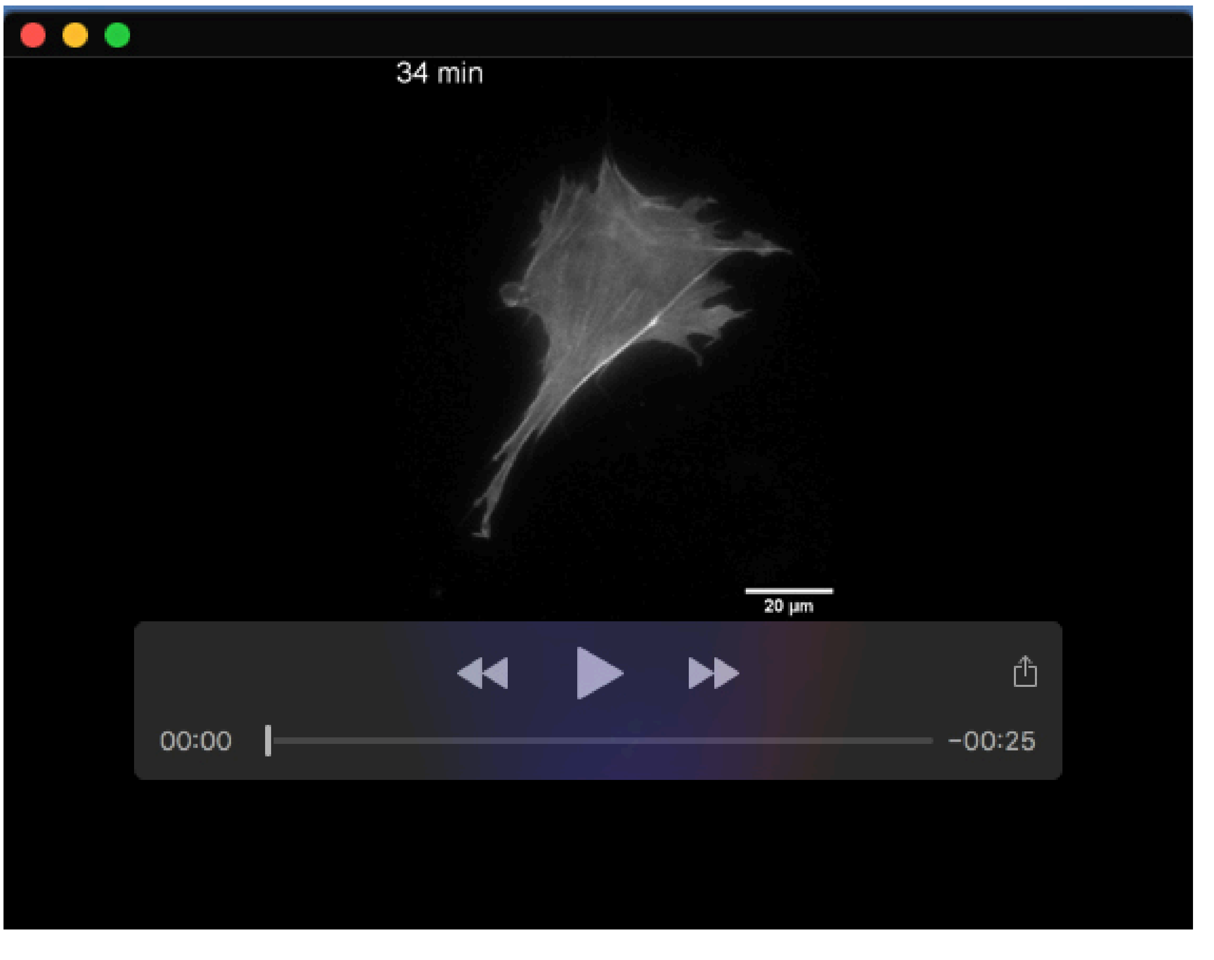

Movie 2. Rearrangement of the actin cytoskeleton following the addition of class I PAK kinase inhibitor. Widefield fluorescence microscopy of a MEF transiently transfected to express mApple-LifeAct, to mark the actin cytoskeleton, responding to the addition of 10 µM G5555. Addition of the drug occurs after 24 minutes, and the point of addition is indicated in the movie. Stills from this movie make up Fig. 1A.

Journal of Cell Science • Supplementary information

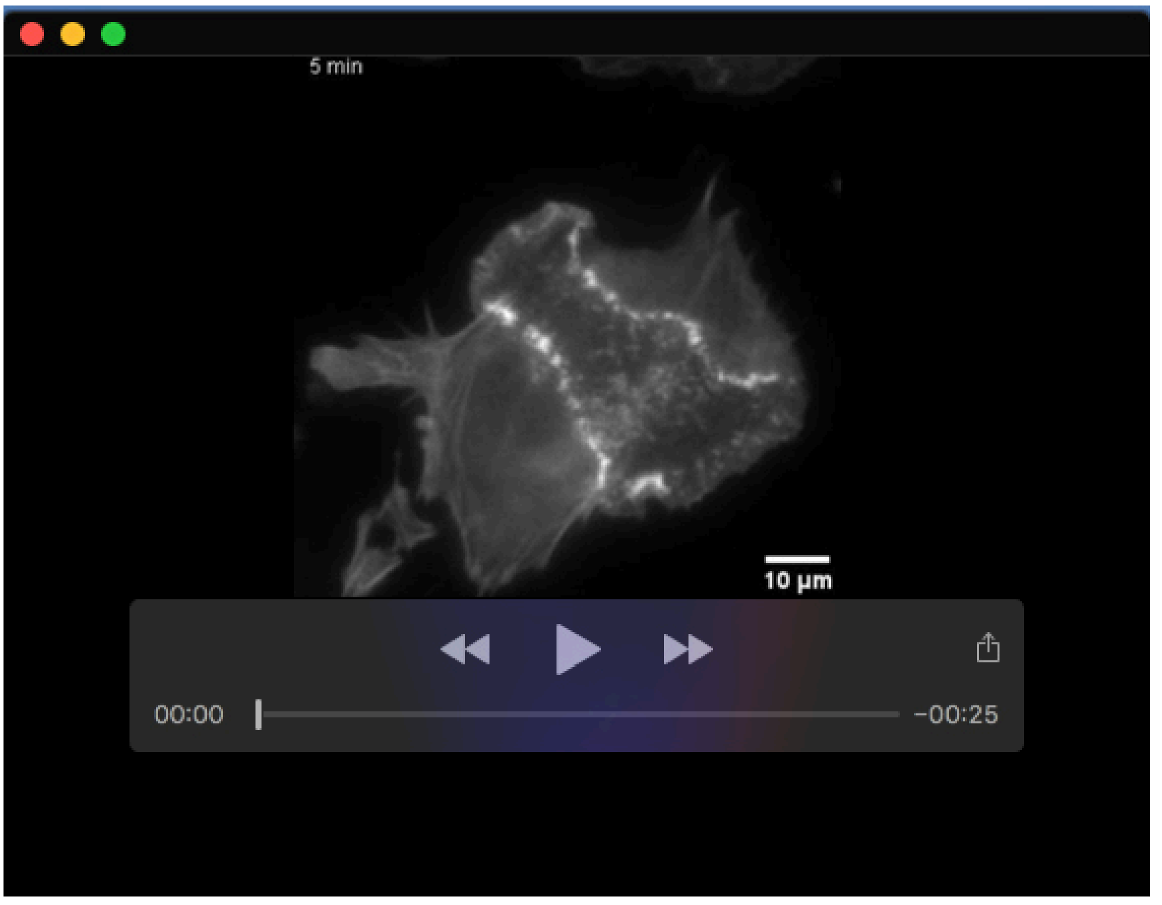

Movie 3. Actin structures that form following class I PAK kinase inhibition are very dynamic, and resemble actin waves. Widefield fluorescence microscopy of a MEF transiently transfected to express mApple-LifeAct, to mark the actin cytoskeleton following the addition of 10 µM G5555. Drug was added at least 1 hour before the start of the movie. Stills from this movie make up Fig. 3C.

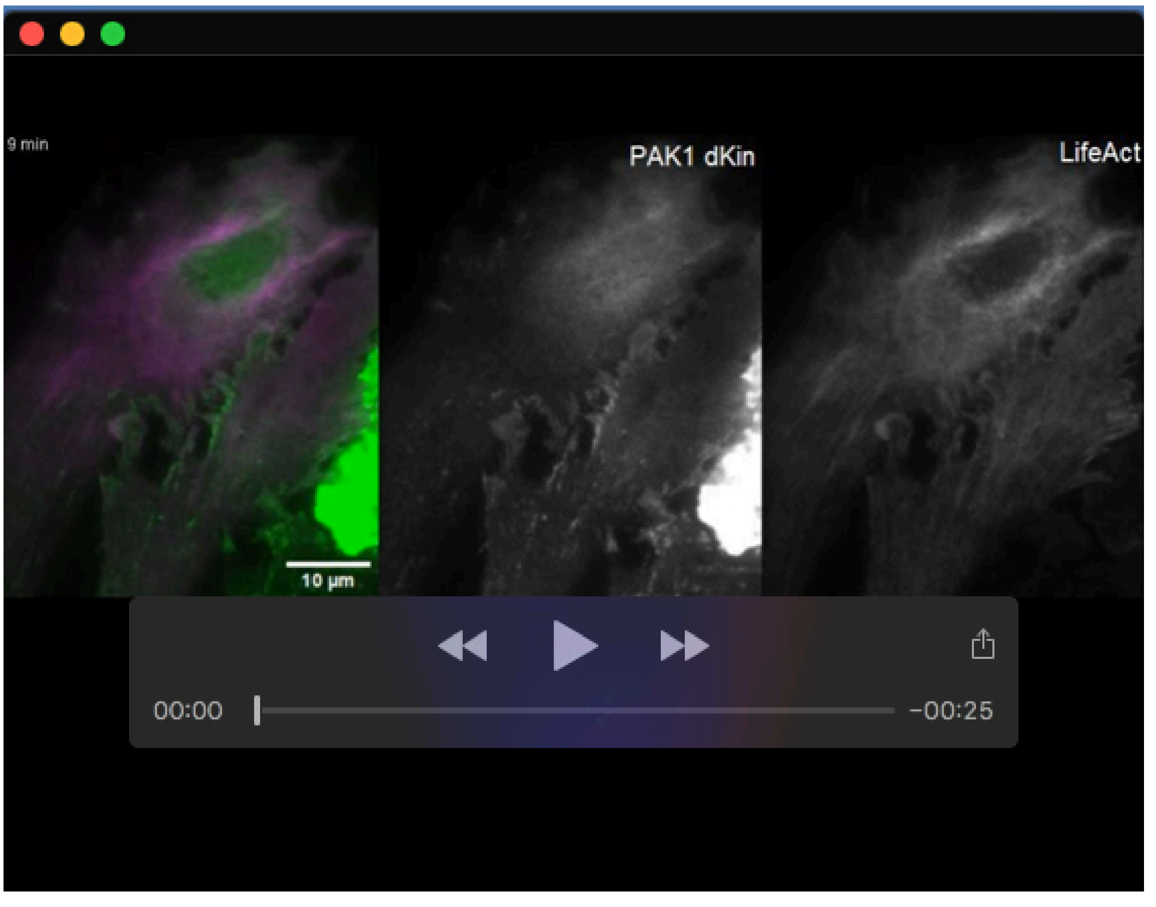

Movie 4. PAK1 Δkin localises within ring of actin, which traverses the ventral surface of cell, resembelling an actin wave. TIRFm of a MEF transiently transfected to express GFP-PAK1 Δkin and mApple LifeAct. Channels are provided separately and as a merge. Stills from this movie make up Fig. 3D.

Journal of Cell Science • Supplementary information

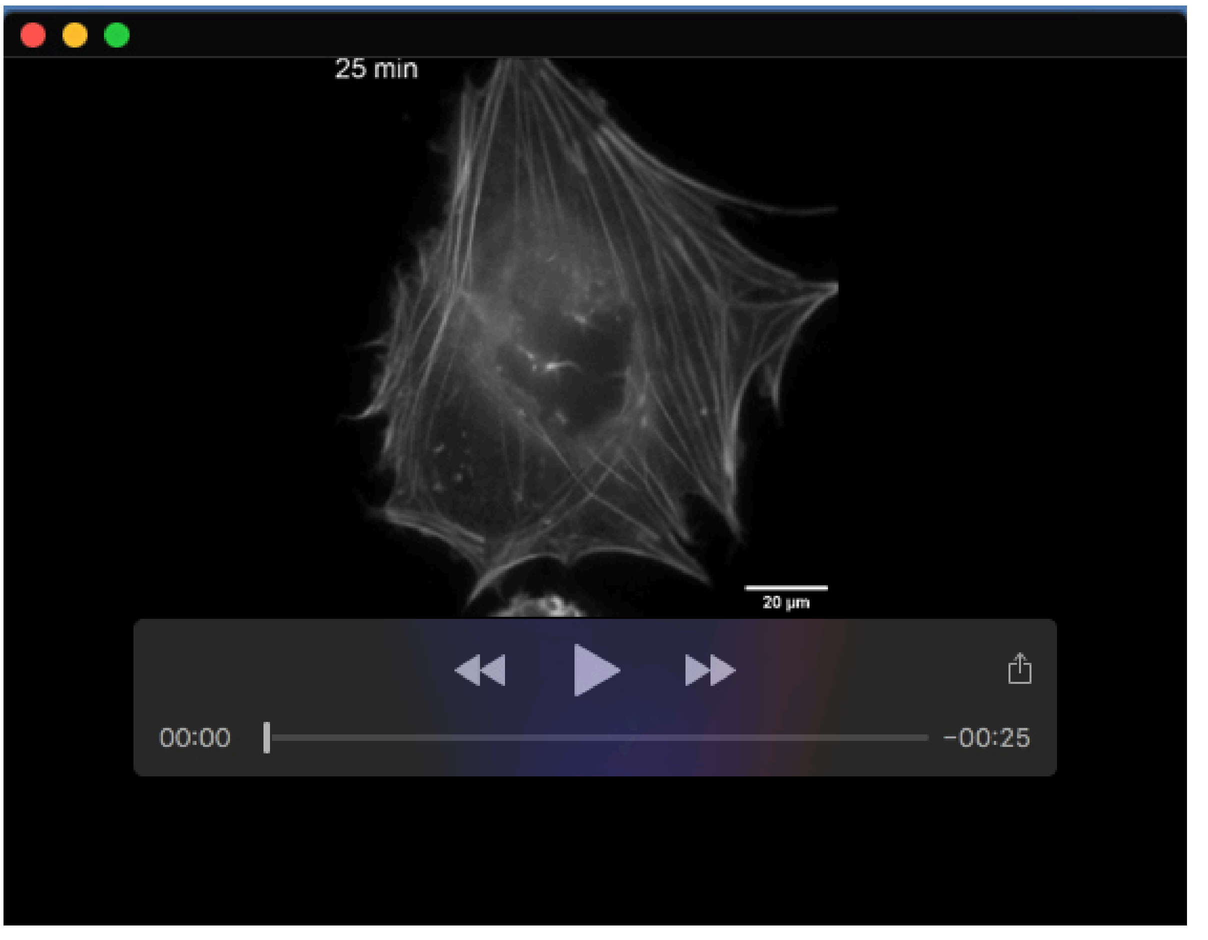

Movie 5. Class I PAK kinase inhibition drives the formation of dramatic travelling actin waves in B16F1 cells. Widefield fluorescence microscopy of a B16F1 cell, transiently transfected to express mEGFP, responding to the addition of 10 µM G5555. Addition of the drug occurs after 60 minutes, and the point of addition is indicated in the movie. Stills from this movie make up Fig. 3E.

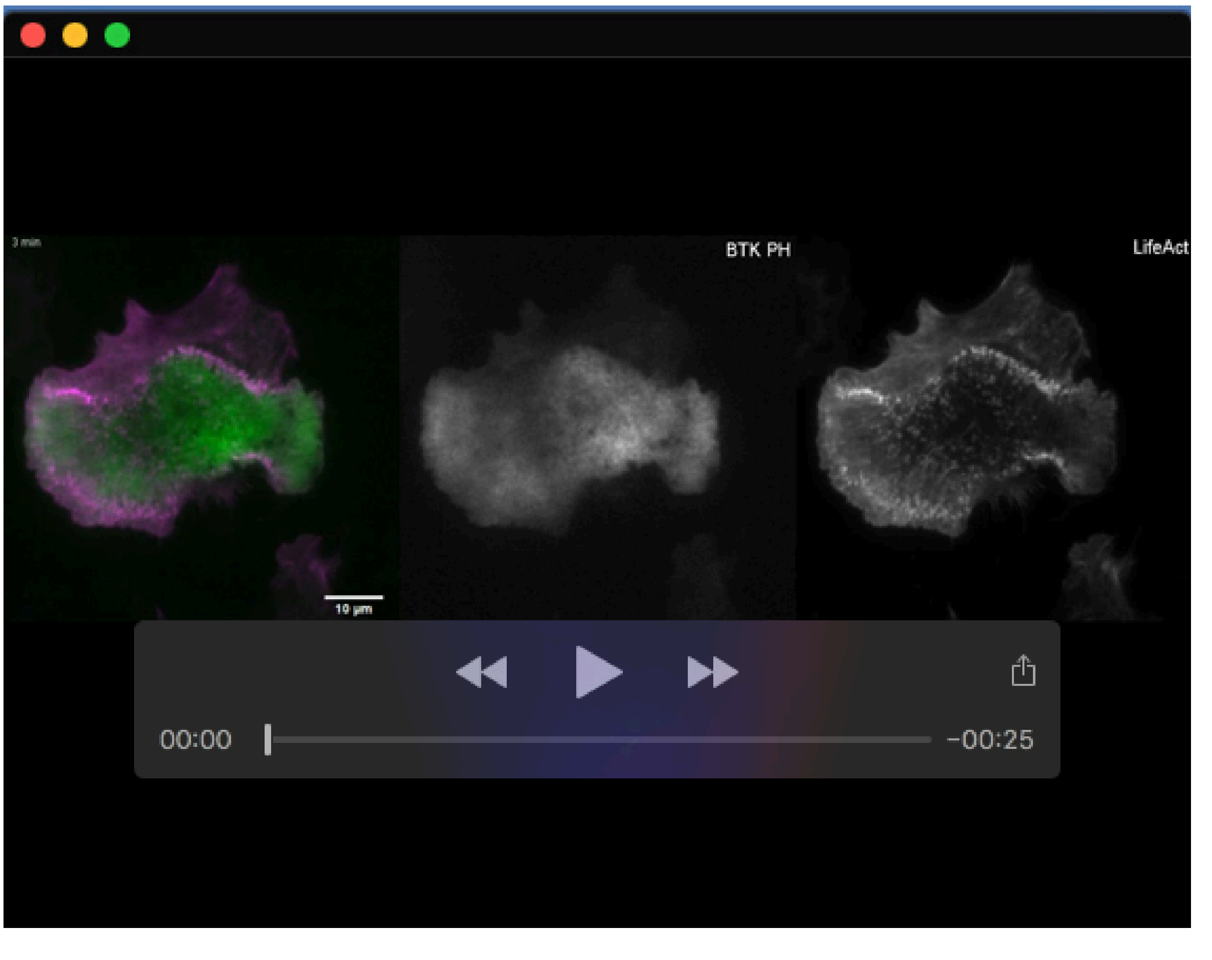

Movie 6. Dynamic actin structures that form following class I PAK kinase inhibition are positive for PIP3. TIRFm of a MEF transiently transfected to express GFP BTK PH to mark PIP3 and mApple LifeAct and treated with 10 µM G5555 atleast 1 hour before imaging. Channels are provided separately and as a merge. Stills from this movie make up Fig. 4A.

Journal of Cell Science • Supplementary information

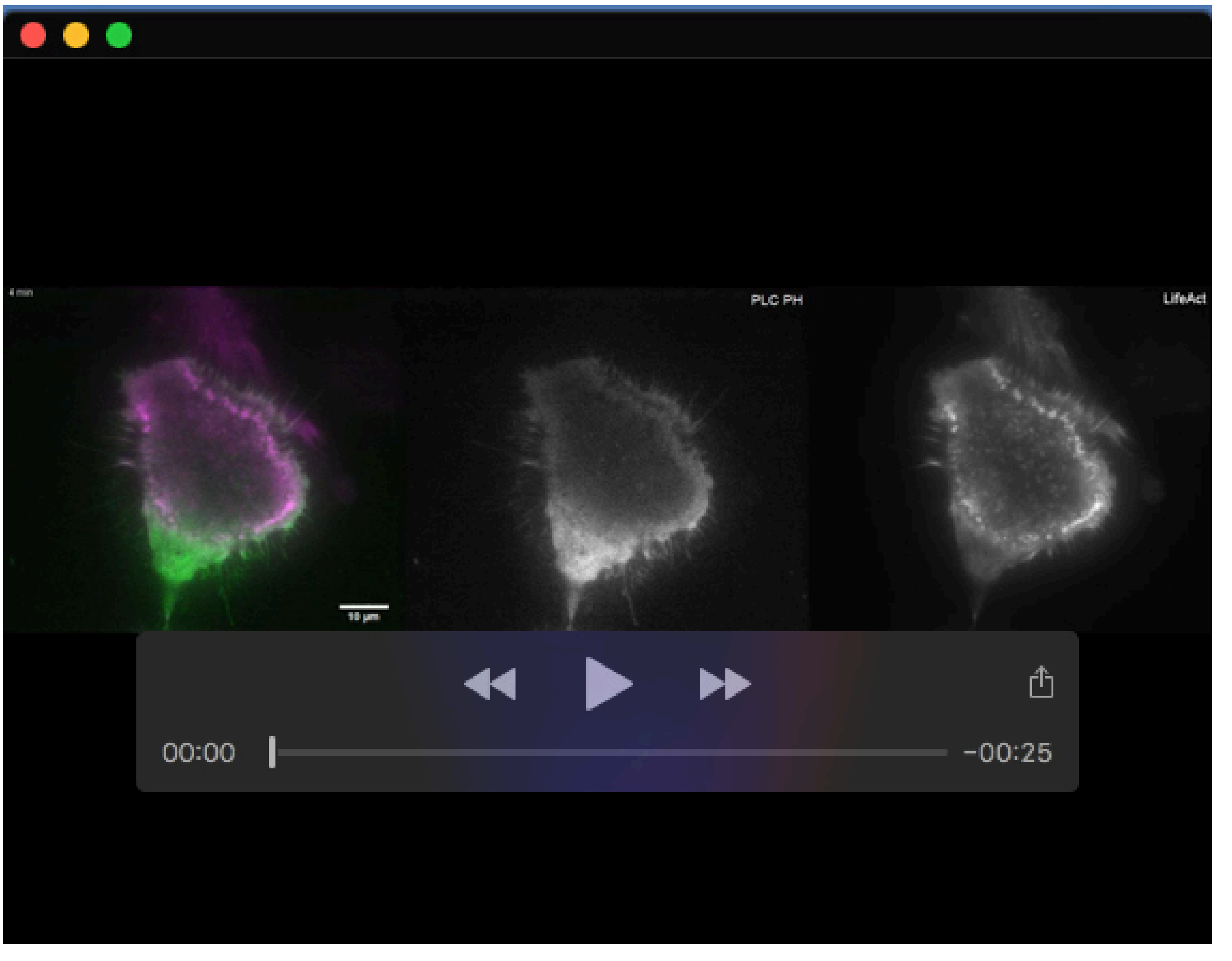

Movie 7. Dynamic actin structures that form following class I PAK kinase inhibition exclude Pi(4,5)P$_2$. TIRFm of a MEF transiently transfected to express mcherry PLC PH to mark Pi(4,5)P2 and mEGFP LifeAct and treated with 10 µM G5555 atleast 1 hour before imaging. Channels are provided separately and as a merge. Stills from this movie make up Fig. 4A.

Journal of Cell Science • Supplementary information
