# Supplementary material for: A role for class I p21-activated kinases in the regulation of the excitability of the actin cytoskeleton
Source: J Cell Sci. 2025 Jun 23;138(12):jcs263763. doi: 10.1242/jcs.263763 (PMC12273633; doi:10.1242/jcs.263763)
Supplement: Supplementary file 1 [file joces-138-263763_review_history.pdf]

A role for class I PAKs in the regulation of the excitability of the actin cytoskeleton

Joe J. Tyler, Anthony Davidson, Megan E. Poxon, Montserrat Llanses Martinez, Pete Hume, Jason S. King and Vassilis Koronakis

DOI: 10.1242/jcs.263763

Editor: Michael Way

Review timeline

Original submission:	4 December 2024
Editorial decision:	7 February 2025
First revision received:	8 May 2025
Accepted:	12 May 2025

Original submission

First decision letter

MS ID#: jcs.263763

MS TITLE: A role for class I PAKs in the regulation of the excitability of the actin cytoskeleton

AUTHORS: Joe J Tyler; Anthony Davidson; Megan E Poxon; Montserrat Llanses Martinez; Pete J Hume; Jason S. King; Vassilis Koronakis

ARTICLE TYPE: Research Article

Dear Dr Tyler,

We have now reached a decision on the above manuscript.

To see the reviewers' reports and a copy of this decision letter, please go to:

As you will see, the reviewers gave favourable reports but raised some points that will require amendments to your manuscript. I hope that you will be able to carry these out because I would like to be able to accept your paper, depending on further comments from reviewers.

Reviewer 1

Advance summary and potential significance to field

This is an interesting study where the authors have sought to understand the actin cytoskeletal consequences of specifically inhibiting PAK kinase activity. They observe that kinase inhibition but not loss of protein leads to an accumulation of PIP3 moieties which they conclude drives the formation of cortactin rich actin rings. The work is well described with high quality imaging and analysis. I have some concerns about the interchangeable use of PAK, class 1 PAK and PAK1 which needs to be resolved. I also have concerns that inhibitor treatments don't appear to have been performed in the presence of a vehicle control. The work has implications for researchers using PAK kinase inhibitors.

© 2025. Published by The Company of Biologists under the terms of the Creative Commons Attribution License (https://creativecommons.org/licenses/by/4.0/).

Comments for the author

1. All figure legends should contain detail about the concentration of inhibitor used and for how long cells were inhibited before being analysed.
2. The figure legends for all of the SFigures are out of sync
3. The conc of inhibitor (10uM) is high for these kind of experiments - can the authors reference prior usage at this concentration
4. Thought the manuscript the authors need to be very clear if they are inhibiting PAK (that would be all 6) - they are inhibiting Group 1 Paks (1-3) or they are specifically inhibiting PAK1 - eg page2line71 - what does G5555 inhibit? What does NVSPAK1 inhibit - this should also be considered in the context of the conc of inhibitor used and any potential off target effects.
5. Line 102 - PAK kinase activity is not inhibited - Group 2 still active (see point 3 above)
6. Figure 1G - Please supply westerns of siRNA knockdown levels
7. Figure 2B - what happens to cell shape when the PAK mutant constructs are overexpressed - does it correlate with ring formation ?
8. Is this effect in Figure 2B specific to PAK1 ? what happens if you overexpress the PBD+_AID of PAK2?
9. Line 152 states AID interaction with Kinase domain required but the PBD+AID construct can induce rings - this needs further clarification
10. Figure 2D - there are multiple bands for B_PIX how was the fold change quantified?
11. Figure 3A can you localise accumulation of endogenous PAK1 at rings in G5555 treated cells ?
12. Do you see cortactin/actin rings in the MiaPa cells?

<u>Reviewer 2</u>

Advance summary and potential significance to field

Review of "A role for class I PAKs in the regulation of the excitability of the actin cytoskeleton" by Tyler et al.

Paks are serine/threonine kinases that are best known as downstream targets of two small GTPases of the Rho family, namely Rac and Cdc42. It has long been assumed that Paks exert their effects on the cortical cytoskeleton via phosphorylation of a handful of key targets such as LIMK and MRLCK which regulate actin assembly and myosin activity, respectively. However, it has also been suspected for some time that Paks may have kinase independent activities and the results obtained for manipulation of Paks in different cell types using different constructs and/or pharmacological inhibitors have been confusing, to say the least.

In the current paper, Tyler et al. make the starting (and startling) observation that pharmacological Pak inhibition results in rapid polarization of MEFs as manifest by formation of a large lamellipodium. After confirming this phenotype with different inhibitors and different cell types, they dive into the mechanisms that explain this surprising result (surprising in the sense that Rac and thus Pak has classically been associated with lamellipodium formation, thus inhibition of Pak would be expected to suppress lamellipodium formation rather than trigger it).

Using a series of complementary experiments and approaches, the authors convincingly demonstrate that this phenotype results from an imbalance of Pak recruitment and Pak kinase activity, leading to the notion that under normal circumstances, the extent to which Pak is activated is normally limited by it's own kinase activity. They further show that part of this effect is mediated through recruitment of BPix, a GEF for Rac and Cdc42. Thus, the authors identify a mechanism for positive feedback via Rac-GTP dependent recruitment of a Rac activator (BPix) coupled to delayed negative feedback via Rac-GTP dependent recruitment of Pak which suppresses additional Rac recruitment via its kinase activity. This scheme is expected to generate cortical excitability and indeed, the authors show that the cells in question generate periodic pulses of F-actin, a hallmark of cortical excitability.

There are studies that describe the discovery of new macromolecular players, and there are studies that put together previously identified players in a manner that provides new insight into how these

© 2025. Published by The Company of Biologists under the terms of the Creative Commons Attribution License (https://creativecommons.org/licenses/by/4.0/).

players work together in the cell. This work represents the latter type of study and as such, it is a major contribution that will be useful both to those seeking to understand what Paks do in the general sense and those who are interested in understanding network interactions within cells. I have a few minor suggestions; these are listed below.

Comments for the author

Figure 1A doesn't really capture the impressive phenotype of Pak inhibition well, especially when compared to movies 1 and 2. The authors should probably just have a single timepoint prior to drug treatment and add two more, including one that shows the fully polarized phenotype (ie one equivalent to the second schematic image in figure 1H).

It wasn't clear what the authors meant by "Identity of cortactin puncta". After reading the relevant section, it seemed more sensible to retitle it "Cortactin puncta resemble podosomes" or "Cortactin puncta resemble podosomes and invadopodia".

The kymograph (which is otherwise excellent) shown in figure 3E should have both the time and space axes labeled with perpendicular bars that allow the reader to relate the image to the amount of elapsed time in one dimension and the amount of space in the other. Additionally, the authors should provide the movie that corresponds to the kymograph so the reader can see the full sequence of cortical excitability development prior to and after drug application. It is possible that one of the supplemental movies were used to generate this kymograph but, if so, this is not indicated in the figure legend for figure 3 (as it was for figure 1).

Legends should be provided for the movies and it should be clear in each case which figure the movies correspond to.

Within the results the demonstration of excitability is lumped in with the "Identity..." work. However, the excitability is one of the major features of this work and arguably more important than the demonstration that the cortactin puncta resemble podosomes. Certainly, the title of the paper gives primacy to the excitability. It would therefore make sense for the authors to subordinate the "identity" to the "excitability" in the results heading.

First revision

Author response to reviewers' comments

Reviewers comments

Reviewer 1: SUMMARY OF THE ADVANCE MADE IN THIS PAPER AND ITS POTENTIAL SIGNIFICANCE TO THE FIELD

This is an interesting study where the authors have sought to understand the actin cytoskeletal consequences of specifically inhibiting PAK kinase activity. They observe that kinase inhibition but not loss of protein leads to an accumulation of PIP3 moieties which they conclude drives the formation of cortactin rich actin rings. The work is well described with high quality imaging and analysis. I have some concerns about the interchangeable use of PAK, class 1 PAK and PAK1 which needs to be resolved. I also have concerns that inhibitor treatments don't appear to have been performed in the presence of a vehicle control. The work has implications for researchers using PAK kinase inhibitors.

We thank the reviewer for their neat summary, positive assessment, and constructive suggestions. The reviewer is completely correct that consistency when referring to PAK, class 1 PAK etc has been an issue and we hope this has been resolved (see below).
Alongside new data for the overexpression of PAK2 Δkin we think these changes have really improved the manuscript.

© 2025. Published by The Company of Biologists under the terms of the Creative Commons Attribution License (https://creativecommons.org/licenses/by/4.0/).

The point about a vehicle control is also a very good one. We have included a DMSO control in figure 1F to demonstrate that cells do not form cortactin/ actin rings in the presence of DMSO alone. This should have always been included and so we thank the reviewer for their concerns. The lipid bead pull downs (fig 2D) were performed with a DMSO control, although this was only indicated in the materials and methods. The figure and figure legend have been altered to make this clear. Similarly, the 'No gradient' and 'gradient' conditions in the chemotaxis data (fig 5B) were performed in the presence of 1 equivalent volume of DMSO. This was not indicated anywhere in the text and this has been amended and we apologise for this oversight.

We have also included an image of MEFs treated with one equivalent volume of DMSO (1:1000) fixed and stained for actin and cortactin (equivalent of figure 1E) (supp. 1E) to demonstrate there is no upregulation of lamellipodia formation in this condition and referenced this in the text (line135)

SUGGESTIONS TO AUTHORS

1. All figure legends should contain detail about the concentration of inhibitor used and for how long cells were inhibited before being analysed.

Very good point, all figure legends have been amended.

2. The figure legends for all of the SFigures are out of sync

Thankyou for spotting this, this was due to a mistake whilst trying to get the supplementary figure file small enough following transfer from biorxv and has been amended.

3. The conc of inhibitor (10uM) is high for these kind of experiments - can the authors reference prior usage at this concentration

Relatively high concentrations were used to remain consistent with our previous salmonella work (Davidson et al 2021). We have mentioned this in the text as well as referring to a new dilution series which shows that a similar effect can be observed at lower concentrations of G5555 (Supp1F) (lines 149-152).

4. Thought the manuscript the authors need to be very clear if they are inhibiting PAK (that would be all 6) - they are inhibiting Group 1 Paks (1-3) or they are specifically inhibiting PAK1
- eg page2line71 - what does G5555 inhibit? What does NVSPAK1 inhibit
- this should also be considered in the context of the conc of inhibitor used and any potential off target effects.

Thankyou for this great point: consistency here has been a weak point of the manuscript. We have changed all mentions of PAK to be more specific and been clearer about what the specific inhibitors target (class I PAKs G5555, PAK1 NVSPAK1). We have chosen to largely use 'class I PAK kinase inhibition' throughout except in the case of overexpression of PAK1 or PAK2 constructs. As we discuss below, we have now included quantification of the effect of the overexpression of PAK2 Δkin in MEFs on cortactin ring formation and we think that this further justifies treating the effect as one of class I PAK kinase inhibition, not just PAK1 kinase inhibition.

As for concerns about off target effects at these relatively high concentrations of drugs: we share them and that is why we include NVSPAK1 and G5555 together in figure 1B-D. The logic being that different classes of molecules, with different modes of action, are less likely to share off target effects. We think that this, coupled with the overexpression data in figure 2, is sufficient to show that the formation of cortactin rings and resulting changes in cell morphology is specific to inhibition of class I PAK kinase activity and is not an off-target effect. We also now show ring formation following the application of a lower concentration of G5555 (Supp 1F).

5. Line 102 - PAK kinase activity is not inhibited - Group 2 still active (see point 3 above)

Again, good point and this has been changed to be more specific.

6. Figure 1G - Please supply westerns of siRNA knockdown levels

Quantification of PAK 1 and 2 levels following double KD by siRNA has been provided along

© 2025. Published by The Company of Biologists under the terms of the Creative Commons Attribution License (https://creativecommons.org/licenses/by/4.0/).

with a blot demonstrating how we decided which band related to which PAK. Full image of all western blots are now included in new supplemental figure including blot that quantification was performed on.
Unfortunately, it turns out neither of the first authors were able to visualise βPIX in MEF extracts and so no quantification can be provided. I'm afraid there was a bit of a mix up and we both thought that the other had successfully performed the blots. We have made this clear in the text (lines 247-249). We hope that the reviewer will agree that since we see an effect on ring formation following the overexpression of βPIX as well as following treatment with siRNA that there is enough supporting evidence justify keeping the data in. Especially as we have previously reported an effect on salmonella infection following treatment of the same cell line with the same oligos and protocol (Davidson et al 2021: A kinase-independent function of PAK is crucial for pathogen-mediated actin remodelling).

7. Figure 2B - what happens to cell shape when the PAK mutant constructs are overexpressed - does it correlate with ring formation?
Good point, yes: when ring formation is present, cells form large lamellipodia. We have mentioned in the text that cells overexpressing PAK mutant constructs generate large lamellipodial protrusions and have included a representative image of cell expressing Pak1 dkin, (Supp 2A and lines 182-185).

8. Is this effect in Figure 2B specific to PAK1 ? what happens if you overexpress the PBD+_AID of PAK2?
Really good point that fits nicely with the call for better consistency when referring to PAK/class I PAKs. Overexpression of PAK2 ΔKin leads to a similar increase in cortactin ring formation, demonstrating that this effect is not specific to the PAK1 inhibition.
Included data for PAK2 dkin and image of its localisation and referenced in the text (Fig. S2 B,C and lines 185-187).

9. Line 152 states AID interaction with Kinase domain required but the PBD+AID construct can induce rings - this needs further clarification
We mean that overexpressed PBD-AID can interact with the kinase domain of endogenous PAK in an intermolecular interaction. We have added an extra line of text to make this more explicit (214-217).
10. Figure 2D - there are multiple bands for B_PIX how was the fold change quantified?
We think that the multiple bands represent different splice variants. The fold change was calculated by including all bands. This has been clarified in the Methods (line 529- 530)

11. Figure 3A can you localise accumulation of endogenous PAK1 at rings in G5555 treated cells?
We can observe class I PAKs at rings. We have included an image of a MEF treated with 10 μM G5555 for an hour before fixation and labelling with a PAK 1,2,3 antidbody. The localisation we observe is very similar to what we see with overexpressed proteins. We don't see clear localisation to focal adhesions in our endogenous staining however this could be because this image was acquired by widefield fluorescent microscopy and not TIRFm.
We have included an image of a MEF stained with PAK 1,2,3 antibody Fig S3A and referred to it in the text (lines 266-267).

12. Do you see cortactin/actin rings in the MiaPa cells?

This is a good point that we hadn't thought to clarify. No, we don't see rings in MiaPacca and we now mention this in the text (lines 403-405). The spreading phenotype described for Miapaccas in Fig S5D is consistent with a change in the excitability of the cytoskeleton (eg the Devreotes lab has described similar spreading events following changes in the position of the threshold that determines when and where PIP3 is produced) but we haven't looked at this cell line in detail and have predominantly used them as they perform pi3k independent macropinocytosis.

This raises something that I would have liked to go into in more detail in the paper which is that I think that the exact response of any given cell type to class I PAK kinase inhibition depends on exactly how that cell responds to the formation of PIP3 at the plasma membrane.

© 2025. Published by The Company of Biologists under the terms of the Creative Commons Attribution License (https://creativecommons.org/licenses/by/4.0/).

Basically, what I think is comparable across cell lines is a pi3k dependent change in the state of the cytoskeleton upon class I PAK kinase inhibition.

Exactly what that change looks like is quite cell line specific. In my (J Tyler) experience it usually involves the formation of some kind of actin wave; but these are very heterogeneous structures. I think that a coherent study of the effect of class I PAK kinase inhibition across cell types could be an interesting way to explore the excitable networks that control PIP3 production and examine how PIP3 production can influence diverse (and sometimes incompatible) cellular functions. We have tried to focus mostly on MEFs (and B16F1s) to not complicate an already complicated story.

Reviewer 2: SUMMARY OF THE ADVANCE MADE IN THIS PAPER AND ITS POTENTIAL SIGNIFICANCE TO THE FIELD

Review of "A role for class I PAKs in the regulation of the excitability of the actin cytoskeleton" by Tyler et al.

Paks are serine/threonine kinases that are best known as downstream targets of two small GTPases of the Rho family, namely Rac and Cdc42. It has long been assumed that Paks exert their effects on the cortical cytoskeleton via phosphorylation of a handful of key targets such as LIMK and MRLCK which regulate actin assembly and myosin activity, respectively. However, it has also been suspected for some time that Paks may have kinase independent activities and the results obtained for manipulation of Paks in different cell types using different constructs and/or pharmacological inhibitors have been confusing, to say the least.

In the current paper, Tyler et al. make the starting (and startling) observation that pharmacological Pak inhibition results in rapid polarization of MEFs as manifest by formation of a large lamellipodium. After confirming this phenotype with different inhibitors and different cell types, they dive into the mechanisms that explain this surprising result (surprising in the sense that Rac and thus Pak has classically been associated with lamellipodium formation, thus inhibition of Pak would be expected to suppress lamellipodium formation rather than trigger it).

Using a series of complementary experiments and approaches, the authors convincingly demonstrate that this phenotype results from an imbalance of Pak recruitment and Pak kinase activity, leading to the notion that under normal circumstances, the extent to which Pak is activated is normally limited by it's own kinase activity. They further show that part of this effect is mediated through recruitment of BPix, a GEF for Rac and Cdc42. Thus, the authors identify a mechanism for positive feedback via Rac-GTP dependent recruitment of a Rac activator (BPix) coupled to delayed negative feedback via Rac-GTP dependent recruitment of Pak which suppresses additional Rac recruitment via its kinase activity. This scheme is expected to generate cortical excitability and indeed, the authors show that the cells in question generate periodic pulses of F-actin, a hallmark of cortical excitability.

There are studies that describe the discovery of new macromolecular players, and there are studies that put together previously identified players in a manner that provides new insight into how these players work together in the cell. This work represents the latter type of study and as such, it is a major contribution that will be useful both to those seeking to understand what Paks do in the general sense and those who are interested in understanding network interactions within cells. I have a few minor suggestions; these are listed below.

We thank the reviewer for this very clear and encouraging summary. I (J Tyler) wish I'd written it as clearly as that myself. We thank the reviewer for their constructive suggestions, we have followed them and think that they improve the presentation of the data. We particularly thank the reviewer for what we have taken as an invitation to be more explicit in the text about changes to the excitability of the cytoskeleton following class I PAK kinase inhibition. We think that changes to the text and subheadings have highlighted this aspect of the work further.

SUGGESTIONS TO AUTHORS

© 2025. Published by The Company of Biologists under the terms of the Creative Commons Attribution License (https://creativecommons.org/licenses/by/4.0/).

Figure 1A doesn't really capture the impressive phenotype of Pak inhibition well, especially when compared to movies 1 and 2. The authors should probably just have a single timepoint prior to drug treatment and add two more, including one that shows the fully polarized phenotype (ie one equivalent to the second schematic image in figure 1H).

We have changed the montage to include just a single timepoint before the addition of the drug and three timepoints after and I think it is much improved. We have included three timepoints after the addition of the drug as I think it is important to capture just how quickly the cytoskeleton responds (therefore the 11 min timepoint) but the shape change is so dramatic that it is hard to recognise that it is the same cell without an intermediate image (22min) between it and the fully polarised phenotype (61min).
Thankyou for the great suggestion.

It wasn't clear what the authors meant by "Identity of cortactin puncta". After reading the relevant section, it seemed more sensible to retitle it "Cortactin puncta resemble podosomes" or "Cortactin puncta resemble podosomes and invadopodia".
Good suggestion, we have changed this subheading.

The kymograph (which is otherwise excellent) shown in figure 3E should have both the time and space axes labeled with perpendicular bars that allow the reader to relate the image to the amount of elapsed time in one dimension and the amount of space in the other. Additionally, the authors should provide the movie that corresponds to the kymograph so the reader can see the full sequence of cortical excitability development prior to and after drug application. It is possible that one of the supplemental movies were used to generate this kymograph but, if so, this is not indicated in the figure legend for figure 3 (as it was for figure 1).

Altered figure to include indication of time and distance covered by kymograph and to indicate which axes is which to aid interpretation.

Legends should be provided for the movies and it should be clear in each case which figure the movies correspond to.

Very good point, legends have now been provided.

Within the results the demonstration of excitability is lumped in with the "Identity..." work. However, the excitability is one of the major features of this work and arguably more important than the demonstration that the cortactin puncta resemble podosomes. Certainly, the title of the paper gives primacy to the excitability. It would therefore make sense for the authors to subordinate the "identity" to the "excitability" in the results heading.

This is a good point. Our understanding of this project has continued to evolve while preparing the manuscript and we should have been more consistent about the importance of the changes to the excitability of the cytoskeleton throughout as we came to understand them. We have restructured the original subheadings as the style was inconsistent with the headings throughout the manuscript. Now the two sections are separate. The subheading referring to the dynamics of the cortactin rings has been changed to make it clear that we are discussing the excitability of the cytoskeleton. We have inserted another two lines of text (299-302) to try to make the point of this section more explicit.

Second decision letter

MS ID#: jcs.263763R1

MS Title: A role for class I PAKs in the regulation of the excitability of the actin cytoskeleton

© 2025. Published by The Company of Biologists under the terms of the Creative Commons Attribution License (https://creativecommons.org/licenses/by/4.0/).

Authors: Joe J Tyler; Anthony Davidson; Megan E Poxon; Montserrat Llanses Martinez; Pete J Hume; Jason S. King; Vassilis Koronakis

Article Type: Research Article

Dear Dr Tyler,

I feel that there is no need to return the manuscript to the original reviewers as you have sufficiently addressed their comments. I am therefore, happy to tell you that your manuscript has been accepted for publication in Journal of Cell Science, pending standard publication integrity checks.

© 2025. Published by The Company of Biologists under the terms of the Creative Commons Attribution License (https://creativecommons.org/licenses/by/4.0/).
